# Antitumor Effects of Carvacrol and Thymol: A Systematic Review

**DOI:** 10.3389/fphar.2021.702487

**Published:** 2021-07-07

**Authors:** Laeza Alves Sampaio, Lícia Tairiny Santos Pina, Mairim Russo Serafini, Débora dos Santos Tavares, Adriana Gibara Guimarães

**Affiliations:** ^1^ Graduate Program of Applied Sciences to Health, Federal University of Sergipe, Lagarto, Brazil; ^2^ Graduate Program in Health Sciences, Federal University of Sergipe, Aracaju, Brazil; ^3^ Department of Pharmacy, Federal University of Sergipe, São Cristóvão, Brazil; ^4^ Department of Health Education, Federal University of Sergipe, Lagarto, Brazil

**Keywords:** carvacrol, thymol, cancer, antitumor, anticancer

## Abstract

**Background:** It is estimated that one in five people worldwide faces a diagnosis of a malignant neoplasm during their lifetime. Carvacrol and its isomer, thymol, are natural compounds that act against several diseases, including cancer. Thus, this systematic review aimed to examine and synthesize the knowledge on the antitumor effects of carvacrol and thymol.

**Methods:** A systematic literature search was carried out in the PubMed, Web of Science, Scopus and Lilacs databases in April 2020 (updated in March 2021) based on the PRISMA 2020 guidelines. The following combination of health descriptors, MeSH terms and their synonyms were used: carvacrol, thymol, antitumor, antineoplastic, anticancer, cytotoxicity, apoptosis, cell proliferation, *in vitro* and *in vivo*. To assess the risk of bias in *in vivo* studies, the SYRCLE Risk of Bias tool was used, and for *in vitro* studies, a modified version was used.

**Results:** A total of 1,170 records were identified, with 77 meeting the established criteria. The studies were published between 2003 and 2021, with 69 being *in vitro* and 10 *in vivo.* Forty-three used carvacrol, 19 thymol, and 15 studies tested both monoterpenes. It was attested that carvacrol and thymol induced apoptosis, cytotoxicity, cell cycle arrest, antimetastatic activity, and also displayed different antiproliferative effects and inhibition of signaling pathways (MAPKs and PI3K/AKT/mTOR).

**Conclusions:** Carvacrol and thymol exhibited antitumor and antiproliferative activity through several signaling pathways. *In vitro*, carvacrol appears to be more potent than thymol. However, further *in vivo* studies with robust methodology are required to define a standard and safe dose, determine their toxic or side effects, and clarify its exact mechanisms of action.

This systematic review was registered in the PROSPERO database (CRD42020176736) and the protocol is available at https://www.crd.york.ac.uk/prospero/display_record.php?RecordID=176736.

## Introduction

It is estimated that one in five people worldwide faces the diagnosis of some malignant neoplasm during their lifetime, and the number of people with cancer is forecast to double by the year 2040 (World Health Organization, 2020). In fact, cancer is a major global public health problem, and it is one of the four main causes of premature death (before 70 years old) in most countries, resulting in 8.8 million deaths per year (National Cancer Institute, 2019). The antineoplastic agents available on the market have different mechanisms of action that impair cell proliferation and/or cause cell death, thereby increasing patient survival rate (Powell et al., 2014; Lee and Park, 2016). However, the toxicity and side effects of many treatments can worsen the quality of life of these individuals (Weingart et al., 2018; Hassen et al., 2019; Lu et al., 2019). Thus, despite being the subject of research for many years, cancer still remains a major concern and an important area of study in the search for a cure.

There is, therefore, an ongoing search for substances that can be used to develop more effective treatments, with less side effects, to use against cancer; one promising group of substances are natural products (NPs). There are many medicinal plants whose pharmacological properties have already been described and scientifically proven (Nelson, 1982; Mishra and Tiwari, 2011; Carqueijeiro et al., 2020). However, the enormous diversity of nature still holds many plant compounds without sufficient studies, particularly in the oncology area (Gordaliza, 2007; Asif, 2015). Historically, secondary plant metabolites have made important contributions to cancer therapy, such as, the vinca alkaloids (vinblastine and vincristine) and the paclitaxel terpene that was obtained from the *Taxus brevifolia* Nutt. species (Martino et al., 2018). More recently, other compounds, such as perillyl alcohol and limonene -monoterpenes found in aromatic plant species, have been widely studied due to their antitumor potential, and have been included in clinical phase studies (Shojaei et al., 2014; Arya and Saldanha, 2019).

In this context, carvacrol (5-isopropyl-2-methylphenol) and its thymol isomer (2-isopropyl-5-methylphenol), classified as natural multi-target compounds, deserve attention. Both are monoterpenoid phenols, the main components present in essential oils obtained from several plant species of the Lamiaceae and Verbenaceae families, such as oregano (*Origanum vulgare* L.), thyme (*Thymus vulg*aris L.) and “alecrim-da-chapada” (*Lippia gracilis*) (Santos et al., 2016; Salehi et al., 2018; Sharifi-Rad et al., 2018; Baj et al., 2020), which have already been reported to exhibit beneficial effects against many diseases (Silva et al., 2018), including cancer (Elbe et al., 2020; Pakdemirli et al., 2020). In addition, these compounds present anti-inflammatory (Li et al., 2018; Chamanara et al., 2019) and antioxidant (Arigesavan and Sudhandiran, 2015; Sheorain et al., 2019) activities that enable the reduction of inflammation and an increase in enzymatic and non-enzymatic antioxidants in the tumor environment (Gouveia et al., 2018). Hence, this systematic review aims to examine and synthesize knowledge about the antitumor and antiproliferative effect of carvacrol and thymol, as well as to report the main mechanisms of action already described for the two compounds against cancer. to provide guidance for future research.

## Methods

### Question and PICOS Strategy

The purpose of this systematic review was to answer the following question: Do carvacrol and thymol exhibit an anti-tumor effect on cancer cells (*in vitro*) or in animal models of cancer? The review followed the guidelines of the Preferred Reporting Items for Systematic Reviews and Meta-Analyses (PRISMA) (Page et al., 2021).

A PICOS strategy (patient or pathology, intervention, control, and other outcomes and type of study) was used based on: P: Animals with cancer or tumor cells; I: Treatment with carvacrol or thymol; C: No treatment, healthy cells or placebo (vehicle); O: Cytotoxic and antitumor effects, induction of apoptosis and inhibition of proliferation; S: Pre-clinical studies *in vitro* and *in vivo*.

### Data Sources and Literature Search

The research was carried out in the databases PubMed, Web of Science, Scopus and Lilacs in April 2020 (updated in March 2021) using a combination of health descriptors, MeSH terms and their synonyms, such as antitumor, antineoplastic, anticancer, cytotoxicity, apoptosis, cell proliferation, *in vitro*, *in vivo*, carvacrol or 5-isopropyl-2-methylphenol and thymol or 5-methyl-2-propan-2-ylphenol (Supplementary Table S1, contains a complete list of these search terms).

### Study Selection and Eligibility Criteria

Two independent reviewers (L.A.S. and L.T.S.P.) analyzed the research results and selected potentially relevant studies after reading their title and abstract, using the systematic review application, Rayyan (Ouzzani et al., 2016). We used the Kappa statistical test to measure the inter-rater reliability (Landis and Koch, 1977). Disagreements were resolved through a consensus between the reviewers, and the decision was supported by the assistance of a third reviewer when necessary (AGG). The following inclusion criteria were applied: Administration of carvacrol or pure thymol vs. placebo; *in vitro* studies of cancer cell lines, *in vivo* study of animals with cancer; cytotoxic effect, antitumor effect, inhibition of proliferation and apoptosis and experimental studies (*in vitro* and *in vivo*). The exclusion criteria were: experiments with derivatives of the carvacrol or thymol, association of the two compounds with other substances or in mixtures composing essential oils and extracts, animals with other diseases in addition to cancer, review articles, meta-analyses, abstracts, conference articles, editorials/letters and case reports. A manual search of the reference lists of all selected studies was also conducted, in order to identify additional primary studies for inclusion.

### Data Extraction and Risk of Bias Assessment

We extracted the following data from the included articles: author, year, country, data about the monoterpene (source, obtention method), concentration and/or dose, type of animal or cell line, results (cytotoxicity, cell proliferation, apoptosis, cell cycle, histology), proposed mechanisms involved in the antitumor effect and conclusion. The authors of the included studies were contacted when necessary (whenever any data or article was not available).

The SYRCLE Risk of Bias tool was used to assess the risk of bias of all *in vivo* experimental studies (Hooijmans et al., 2014). We analyzed the following ten domains: sequence generation, allocation concealment and random accommodation (selection bias), random accommodation and concealment (performance bias), random evaluation and concealment of results (detection bias), incomplete result data (bias of attrition), selective report of results (report bias) and other sources of bias, such as inappropriate influence from financiers. An adapted protocol of the SYRCLE Risk of Bias tool was used to evaluate the methodological quality of *in vitro* studies, as described by Chan et al. (2017). The methodological quality was classified as low, unclear or high, according to the established criteria (Hooijmans et al., 2014).

### Statistical Analysis

IC_50_ values determined 24 h after the incubation of the studied cells with carvacrol or thymol were compiled, submitted to the standardization of the unit (μM) (Supplementary Table S2) and were expressed as mean ± standard error of the mean (SEM).

## Results

### Study Selection

**FIGURE 1:**
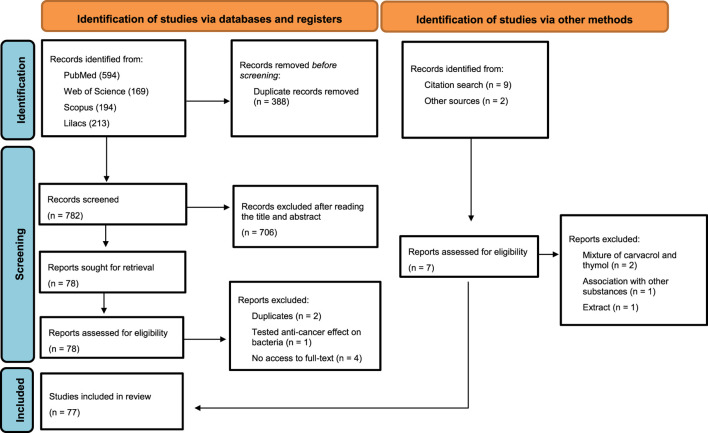
Flowchart of included studies.

The initial search resulted in 1,170 records, of which 594, 169, 194, and 213 were found in PubMed, Web of Science, Scopus and Lilacs, respectively. Of these, 388 were excluded due to duplication. After screening the title and abstract, 706 reports were excluded, and 1 report was sought for retrieval, as it met the criteria after reading the full text, resulting in 77 studies. Of these, three were excluded after reading the full text (two for presenting the same results and one that tested the anti-cancer effect on bacteria) and four for not having access to the full text, resulting in 70 articles. In addition, 9 studies were identified after a manual search of the references and two studies obtained from other sources, but only seven studies were added (two studies were excluded for having a mixture between carvacrol and thymol, one for being associated with other substances, and one that studied the extract of a plant rich in thymol), finally resulting in 77 included studies (Figure 1). There was almost perfect (Landis and Koch, 1977) reliability/agreement (*κ* = 0.813) among the reviewers, after selecting the titles and abstracts.

### Overview of Included Studies

The selected studies were carried out in different countries: India (*n* = 14), China (*n* = 14), Turkey (*n* = 13), the Republic of Korea (*n* = 5), Slovakia (*n* = 5), Iran (*n* = 5), Morocco (*n* = 2), Brazil (*n* = 2), Greece (*n* = 2), Iraq (*n* = 2), The United States of America (*n* = 2), Canada (*n* = 2), Egypt (*n* = 2), Croatia (*n* = 1), Lithuania (*n* = 1), Italy (*n* = 1), Spain (*n* = 1), The United Kingdom (*n* = 1), Peru (*n* = 1), Belgium (*n* = 1). Asia (51.9%) was the continent with the largest number of publications on the subject, followed by Europe (33.7%), the Americas (9.3%) and Africa (5.1%), with a greater trend of publications on this subject in the last three years (Supplementary Figure S1). Among the selected articles, 69 (89.6%) reported *in vitro* experiments and 10 (12.9%) were *in vivo* studies. Likewise, 43, 19, and 15 publications tested only carvacrol, thymol and both compounds, respectively. They were mostly obtained commercially, provided by Sigma Aldrich (*n* = 52), Fluka (*n* = 5), Aldrich Chemical (*n* = 2), Western Chemical (*n* = 1), Agolin SA (*n* = 1), Alfa Aesar (*n* = 1). In only five studies were the compounds isolated from essential oils. Remarkably, ten studies did not report the source of the tested compounds. A detailed description of the included studies is shown in Tables 1 and 2. A narrative summary of the results is presented below, divided into *in vitro* and *in vivo* studies.

**TABLE 1:** Detailed description of the studies that used carvacrol, included in the systematic review.

Model	Concentration/incubation time	Experimental methods for testing IC50 values	Results/targets			Conclusion	Authors (Year), Country
			Increase	Decrease	IC_50_		
Monoterpene carvacrol							
*In vitro* studies							
CO25	1–150 μg/mL	MTT assay	p21^N−ras^	Tumor growth	60 μg/mL–24 h	Carvacrol has a cytotoxic effect and an antiproliferative effect	Zeytinoglu et al. (2003), Turkey
	24, 48, 72 h of incubation			DNA synthesis level			
A549	100–1,000 μM	—	Apoptosis induction	Cell viability	—	Carvacrol may have an anticancer effect and be used as a drug substance to cure cancer	Koparal and Zeytinoglu (2003), Turkey
	24 h of incubation			Cell proliferation			
HepG2	25–900 μmol	—	Cytotoxic effects	DNA damage level	—	HepG2 cells were slightly more sensitive to the effects	Horváthová et al. (2006), Slovakia
Caco-2	24 of incubation						
Leiomyosarcoma	10–4,000 μM	Trypan Blue	Antiproliferative effects	Cell growth	90 μM–24 h	Carvacrol has anticarcinogenic, antiproliferative and antiplatelet properties	Karkabounas et al. (2006), Greece
	24 and 48 h of incubation				67 μM–48 h		
K-562	200–1,000 μM	Trypan blue exclusion	Cytotoxic effects	DNA damage level	220 μM–24 h	Carvacrol has cytotoxic, antioxidant effects and has a protective action against DNA damage	Horvathova et al. (2007), Slovakia
	24 or 48 h of incubation						
P-815	0.004–0.5% v/v	MTT assay	—	—	<0.004% v/v–48 h	Carvacrol is cytotoxic	Jaafari et al. (2007), Morocco
	48 h of incubation						
HepG2	100–1,000 μM	Trypan blue exclusion	Cytotoxic effects	Cell proliferation	HepG2 - 350 μM–24 h	Carvacrol has antiproliferative and antioxidant effects	Slamenová et al. (2007), Slovakia
Caco-2	24 h of incubation				Caco-2 - 600 μM–24 h		
MDA-MB 231	20–100 μM	MTT assay	Apoptosis induction	Cell growth	100 μM–48 h	Carvacrol can be a potent antitumor molecule against breast cancer metastatic cells	Arunasree, (2010), India
			Caspase activation	S-phase cells			
	24 or 48 h of incubation		Sub-stage G0/G1	Mitochondrial membrane potential			
			Cyt C	Bcl-2			
			Bax				
5RP7	0.0002–0.1 mg/mL	MTT assay and Trypan Blue exclusion	Cytotoxic effects	—	5RP7 - 0.04 mg/mL–24/48 h	Carvacrol promoted a cytotoxic effect, induced apoptosis and can be used in cancer therapy	Akalin and Incesu, (2011), Turkey
CO25	24 or 48 h of incubation		Apoptotic cells		CO25–0.1 mg/mL–24 h		
					0.05 mg/mL–48 h		
SiHa	25–500 μg/mL	MTT and LDH assay	Apoptosis induction	Cell proliferation	SiHa - 50 ± 3.89 mg/L	Carvacrol is a potent anticancer compound that exhibits cytotoxic effects and induces the inhibition of cell proliferation in both human cervical cancer cells	Mehdi et al. (2011), India
HeLa	48 h of incubation				HeLa - 50 ± 5.95 mg/L		
HepG2	20–200 μg/mL	CellTiter-Blue^®^ cell viability assay	Cytotoxic effects	Membrane damage	53.09 μg/mL	Carvacrol exhibits antioxidant activity and anticancer effects on cells	Özkan and Erdogan (2011), Turkey
	24 h of incubation		Antiproliferative effects	Cell viability			
P-815	0.05–1.25 μM	MTT assay	Cytotoxic effects	Interruption of cell cycle progression in the S phase	P-815–0.067 μM	Carvacrol showed a cytotoxic effect in all strains tested	Jaafari et al. (2012), Morocco
CEM					CEM - 0.042 μM		
K-562	48 h of incubation				K-562–0.067 μM		
MCF-7					MCF-7 - 0.125 μM		
MCF-7 gem					MCF-7 gem - 0.067 μM		
DBTRG-05MG	200–1,000 μM	—	Generation of ROS	Cell viability	—	Carvacrol was cytotoxic and induced cell death in human glioblastoma cells	Liang and Lu, (2012), China
	24 h of incubation		Caspase-3				
H1299	25–1800 μM	CellTiter-Blue^®^ cell viability assay	MDA	Membrane and DNA damage	380 μM–24 h	Carvacrol exhibited cytotoxic and antioxidant effects	Ozkan and Erdogan (2012), Turkey
	24 and 48 h of incubation		8-OHdG		244 μM–48 h		
B16-F10	Not reported	Trypan blue assay and MTT assay	Cytotoxic effects	Cell viability	550 μM	Carvacrol showed an antitumor effect with moderate cytotoxicity	Satooka and Kubo (2012), United States
	24 h of incubation			Relative melanogenesis			
				Relative melanin cell			
HepG2	0.05–0.4 mmol/L	MTT assay	p-p38	Cell viability	0.4 mmol/L–24 h	Carvacrol caused inhibition of cell proliferation, inhibition of tumor cell growth and induction of apoptosis	Yin et al. (2012), China
	24 h of incubation		MAPK	p-ERK 1/2			
			Caspase-3	Bcl-2			
OC2	200–1,000 μM	—	Generation of ROS	Cell viability	—	Carvacrol exhibited a cytotoxic effect and induced apoptosis in human oral cancer cells	Liang et al. (2013), China
	24 h of incubation		Caspase-3				
MCF-7	140–450 μM	MTT and LDH assay	Caspase-3, -6 and -9	Cell viability	244.7 ± 0.71μM–48 h	Carvacrol induces cytotoxicity and apoptosis in MCF-7 cells and may be a potential chemotherapeutic agent against cancer	Al-Fatlawi and Ahmad (2014), India
	24 and 48 of incubation		Bax	Bcl-2			
			p53				
N2a	10–400 mg/L	—	TAC	—	—	Carvacrol has antioxidant and anticancer properties in N2a cells at concentrations of 200 and 400 mg/L	Aydın et al. (2014), Turkey
	24 h of incubation		TOS				
Caco-2	100–2,500 μM	MTS assay	Apoptosis induction	Cell viability	460 ± 3.6 μM–24 h	Carvacrol exhibited cytotoxic effects and induction of apoptosis	Llana-Ruiz-Cabello et al. (2014), Spain
	24 and 48 h of incubation				343 ± 7.4 μM–48 h		
HepG2	25–1,000 μM	Trypan Blue exclusion and MTT assay	Apoptosis induction	Cell growth	425 μM–24 h	Carvacrol can be used as an anti-tumor molecule against cancer cells	Melusova et al. (2014), Slovakia
	24 h of incubation			SsDNA breaks			
				Oxidative DNA lesions			
HepG2	100–600 μM	—	Cells in G1 phase	S-phase cells	—	Carvacrol caused induction of apoptosis and slowed cell division, resulting in cell death	Melušová et al. (2014), Slovakia
	24 h of incubation						
U87	125–1,000 μM	MTT assay	Apoptosis induction	Cell viability	561.3 μM–24 h	Carvacrol has therapeutic potential for the treatment of glioblastomas by inhibiting TRPM7 channels	Chen et al. (2015), Canada
	24, 48 or 72 h of incubation		Caspase-3	Cell proliferation			
				PI3K/Akt			
				MAPK			
				TRPM7			
				MMP-2			
HCT116	100–900 μmol/L	MTT assay	Apoptosis induction	Cell growth	HCT116–544.4 μmol/L–48 h	Carvacrol can be a promising natural product in the management colon cancer	Fan et al. (2015), China
LoVo	48 h of incubation			Cell migration and invasion			
				Bcl-2			
			Bax	MMP-2 and -9	LoVo - 530.2 μmol/L–48 h		
				Cyclin B1			
				p-ERK			
			p-JNK	p-Akt			
				PI3K/Akt			
				Cell cycle stop in phase G2/M			
AGS	0.01–6 mg/mL	MTT assay	Cytotoxic effects	Cell viability	30 μg/mL–48 h	Carvacrol exhibited a cytotoxic effect against gastric cancer cells	Maryam et al. (2015), Iran
	48 h of incubation						
HL-60	10–200 μM	MTT assay	Apoptosis induction	Cell viability	HL-60–100 μM–24 h	Carvacrol effectively blocked the proliferation of cancer cells *in vitro*	Bhakkiyalakshmi et al. (2016), India
Jurkat	24 h of incubation		Cytotoxic effects	MMP	Jurkat - 50 μM–24 h		
			Generation of ROS	Bcl-2			
			Caspase-3				
			Bax				
Tca-8113	10–80 μM	—	Apoptosis induction	Cell proliferation	—	Carvacrol is a powerful new natural anti-cancer drug for human OSCC	Dai et al. (2016), China
SCC-25	24 and 48 h of incubation			S-Phase cells			
			p21	CCND1			
				CDK4			
				Bcl-2			
			Bax	MMP-2 and -9			
				COX-2			
A549	1–1,000 μM	SRB assay	Antiproliferative effects	—	A549–0.118 ± 0.0012 mΜ–72 h	Carvacrol exhibited antiproliferative and antioxidant effects. In addition, it exhibited more potent cytotoxicity against cells (A549). The cells (Hep3B) were more resistant to treatment and the cells (HepG2) were less sensitive	Fitsiou et al. (2016), Greece
HepG2	72 h of incubation		Cytotoxic effects		HepG2 - 0.344 ± 0.0035 mΜ–72 h		
Hep3B					Hep3B- 0.234 ± 0.017 mΜ–72 h		
PC-3	250–750 μM	CCK-8 Kit	—	Cell viability	PC-3 - 498.3 ± 12.2 μM–24 h	Carvacrol treatment suppresses cell proliferation, migration and invasion, indicating that it has antiprostatic effects *in vitro*	Luo et al. (2016), China
DU 145	24, 48 and 72 h of incubation			Cell proliferation	DU 145–430.6 ± 21.9 μM–24 h		
				Cell migration			
				Wound healing			
				MMP-2			
				PI3K/Akt and MAPK			
				Cell invasion			
				TRPM7			
A549	0–250 μM	—	Cytotoxic effects	Cell viability	—	Carvacrol has cytotoxic activity	Coccimiglio et al. (2016), Canada
	24 h of incubation						
U87	1–10,000 μM	MTT assay	Anticancer activity	—	U87–322 μM–24 h	Carvacrol exerted anticancer and antiproliferative activity with greater effect against the breast cancer cell line	Baranauskaite et al. (2017), Lithuania
MDA-MB 231	24 h of incubation		Antiproliferative activity		MDA-MB 231–199 μM–24 h		
			Antioxidant activity				
HepG2	0.01–0.25 μg/μL	MTT assay	—	Cell viability	48 mg/L–24 h	Carvacrol has therapeutic potential in tumor cells without adverse effects in healthy cells	Elshafie et al. (2017), Italy
	24 h of incubation			Hepatocarcinoma cells			
PC-3	100–800 μM	—	Cytotoxic effects	Cell viability	—	Carvacrol is cytotoxic	Horng et al. (2017), China
	24 h of incubation						
DU 145	10–500 μM	MTT assay	Cytotoxic effects	Cell viability	84.39 μM–24 h	Carvacrol has antiproliferative potential and can act as a chemopreventive agent in prostate cancer	Khan et al. (2017), India
	24 and 48 h of incubation		Apoptosis induction	Cell proliferation	42.06 μM–48 h		
			Caspase-3	Mitochondrial membrane potential			
			Generation of ROS	Cell cycle stop			
			Cells in phase G0/G1	Cells in S and G2/M phases			
SiHa	140–450 μM	MTT assay and LDH	Cytotoxic effects	Cell viability	SiHa - 424.22 μmol –24 h and 339.13 μmol–48 h	Carvacrol exhibited antiproliferative effects and may be a potential chemotherapeutic agent against cancer	Abbas and Al-Fatlawi (2018), Iraq
HepG2	24 and 48 h of incubation		Apoptosis induction	Bcl-2			
			Caspase-3, -6 and -9		HepG2 - 576.52 μmol –24 h and 415.19 μmol –48 h		
			Bax				
			p53				
A375	3.906–1,000 μg/mL	MTT assay	Apoptosis induction	Cell viability	40.41 ± 0.044 μg/mL–24 h	Carvacrol exhibits antiproliferative effects	Govindaraju and Arulselvi (2018), India
	24 of incubation		Sub-G1 phase	Cell growth			
				Bcl-2			
				Cell cycle stop			
				Cells in phase G0/G1 and G2/M			
AGS	10–600 µM	CellTiter-Glo Luminescent cell viability assay	Apoptotic effects	Cell viability	82.57 ± 5.58 µM–24 h	Carvacrol has cytotoxic effects, apoptotic, genotoxic effects and dose-dependent ROS generators	Günes-Bayir et al. (2018), Turkey
	24 h of incubation		Necrosis	Bcl-2			
			Bax				
			Caspase-3 and -9				
			Generation of ROS	GSH levels			
			Genotoxic effect				
AGS	10–600 µM	CellTiter-Glo Luminescent cell viability assay	Cytotoxic effects	Cell viability	82.57 ± 5.5 μM–48 h	Carvacrol inhibited cell proliferation and induced cytotoxicity in cancer cells	Günes-Bayir et al. (2018), Turkey
	48 h of incubation		Apoptosis induction	Bcl-2			
			Bax				
			Caspase-3 e -9				
			Generation of ROS	GSH levels			
			Genotoxic effect				
MCF-7	10–200 μg/mL	MTT assay	—	Cell viability	MCF-7 - 46.5 μg/mL–24 h	Carvacrol has a cytotoxic effect and can cause inhibition of cell growth	Jamali et al. (2018), Iran
MDA-MB 231	24 h of incubation				MDA-MB 231–53 μg/mL– 24 h		
A549	30–300 μM	—	—	Cell viability	-	Carvacrol suppressed cell proliferation and migration and its inhibitory effect was attenuated in NSCLC cells with overexpression of AXL	Jung et al. (2018), Republic of Korea
H460	24 h of incubation			Cell proliferation			
				AXL expression			
				Cell migration			
JAR	50–300 μM	—	Apoptosis induction	Cell proliferation	—	Carvacrol may be a possible new therapeutic agent or supplement for the control of human choriocarcinomas	Lim et al. (2019), Republic of Korea
JEG3	48 h of incubation		Sub-G1 phase	Cell viability			
			Generation of ROS	PI3K/AKT			
			p-JNK	p-ERK1/2			
			p-p38	MMP			
HeLa	100–800 µM	XTT Reduction assay	Induction of cytotoxicity and apoptosis	Cyclin D1	556 ± 39 μM–24 h	Carvacrol can be used to treat cervical cancer, however, it should be avoided during cisplatin chemotherapy	Potočnjak et al. (2018), Croatia
	24 h of incubation		ERK1/2				
			Caspase-9				
			p21				
PC-3	100–800 μM	MTT assay	Cell death	Cell viability	360 μM–48 h	Carvacrol inhibited the ability to invade and migrate PC3 cells and can be considered an anticancer agent	Heidarian and Keloushadi (2019), Iran
	48 h of incubation			Cell proliferation			
				Tumor cell invasion			
				IL-6			
				p-STAT3			
				p-ERK1/2			
				p-AKT			
PC-3	10–500 μM	MTT assay	Apoptosis induction	Cell viability	46.71 μM–24 h	Carvacrol is a chemopreventive agent and has an antiproliferative effect on prostate cancer cells	Khan et al. (2019), India
			Caspases -8 e -9	Cell proliferation			
				Cell migration			
	24 and 48 h of incubation		Generation of ROS	Cell cycle stop at G0/G1	39.81 μM–48 h		
				Cells in S and G2/M phases Bcl-2			
			Bax	Notch-1			
				mRNA Jagged-1			
MCF-7	31.2–500 μg/mL	AlamarBlue^®^ assay	Apoptosis induction	Cell proliferation	MCF-7 - 266.8 μg/mL–48 h	Carvacrol had the most cytotoxic effect among the other components studied	Tayarani-Najaran et al. (2019), Iran
PC-3	48 h of incubation		Cytotoxic effects	Cell viability	PC-3 - >500 μg/mL– 48 h		
DU 145			Bax		DU 145–21.11 μg/mL– 48 h		
PC-3	25–200 μg/mL	—	Cytotoxic effects	Cell viability	—	At the lowest concentration tested (25 μg/ml), carvacrol did not exhibit cytotoxicity to cancer cells	Trindade et al. (2019), Brazil
	24 and 48 h of incubation						
HCT116	25–200 μM	xCELLigence Real-time cell Analysis	—	Cell proliferation	HCT116–92 μM–48 h	Carvacrol has an antiproliferative effect on both cell lines, but is more efficient against HT-29 compared to the HCT116 cell line	Pakdemirli et al. (2020), Turkey
HT-29	48 h of incubation				HT-29–42 μM–48 h		
MCF-7	25–250 μmol/L	MTT and LDH assay	Apoptosis induction	Cell viability	200 μmol/L–24/48 h	Carvacrol can be used in a new approach for the treatment of breast cancer	Mari et al. (2020), India
			Cells in phase G0/G1	Cells in S and G2 phase			
				CDK4 and 6			
	24 and 48 of incubation			Cyclin D1			
			Bax	Bcl-2			
				PI3K/p-AKT			
SKOV-3	100, 200, 400, 600 μM	MTT assay	Apoptosis induction	Cell viability	322.50 µM–24 h	Carvacrol was cytotoxic to the ovarian cancer cell line	Elbe et al. (2020), Turkey
	24 and 48 h of incubation				289.54 µM–48 h		
Kelly	12.5, 25, 50 µM	—	Antiproliferative effects	—	—	Carvacrol can be used to inhibit neuroblastoma cell proliferation	Kocal and Pakdemirli (2020), Turkey
SH-SY5Y	24 h of incubation						
BT-483	25–500 μM	—	Apoptosis induction	Cell viability	—	Carvacrol suppresses breast cancer cells by regulating the cell cycle and the TRPM7 pathway is one of the pharmacological mechanisms	Li et al. (2021), China
BT-474			Cells in G1/G0 phase	S-phase and G2/M cells			
MCF-7	24 h of incubation		Cyclin C, D and E	Cell proliferation			
MDA-MB 231				Cyclin A e B			
MDA-MB 453				CDK 4			
KG1	100, 200, 300, 400 μM	—	Cell death	Cell viability	—	KG1 cell lines were very sensitive to 300 µM carvacrol compared to the HL60 line, while the K562 line showed resistance after 48 h of treatment with 400 µM carvacrol	Bouhtit et al. (2021), Belgium
K-562	24 and 48 h of incubation						
HL-60							

Model	Dose	Results/targets		Conclusion	Authors (Year), Country
		Increase	Decrease		
*In vivo* studies					
Chemical carcinogenesis induced by B [a]P in male wistar rats	20 mL of carvacrol (*ε* = 976 mg/mL) mixed with 200mg of B [a]P	Anticarcinogenic effects	30% tumor incidence	Carvacrol has anticarcinogenic, antiproliferative and antiplatelet properties	Karkabounas et al. (2006), Greece
		Animal survival time	B [a]P carcinogenic potency		
Male wistar rats induced with hepatocarcinogenesis providing 0.01% DEN through drinking water for 16 weeks	Pre-treatment: (15 mg/kg b.wt) of carvacrol orally one week before DEN administration and up to 16 weeks	Final body weight	Pre-treatment: Number of nodules; neoplastic transformations; liver weight	Carvacrol has the ability to cause apoptosis in cancer cells and has a potent elimination of free radicals and antioxidant activities	Jayakumar et al. (2012), India
	Post-treatment: (15 mg/kg b.wt) of carvacrol orally for 6 weeks after administration of DEN for 10 weeks	Chemopreventive effect apoptosis induction	Post-treatment: Persistent but tiny nodules; architecture;		
		GPx, GR, GSH, SOD, CAT	Likely to spread through intrahepatic veins		
			AST, ALT, ALP, LDH, cGT		
Liver carcinogenesis chemically induced by NDEA in male wistar rats orally (dissolved in 0.9% normal saline), in a dose of 20 mg/kg body weight, five times a week, for 6 weeks	15 mg/kg of carvacrol orally, five times a week for 15 weeks, after NDEA administration for 6 weeks	Antiproliferative effect	AFP	Carvacrol may have an antitumor effect through its antiangiogenic capacity, antiproliferative effect and apoptotic activity against tumor cells *in vivo*	Ahmed et al. (2013), Egypt
		Apoptosis induction	VEGF		
		Marked improvement in histological characteristic of liver tissue	AFU		
			GGT		
Male wistar rats induced with hepatocarcinogenesis providing 0.01% DEN through drinking water for 16 weeks	Pre-treatment: (15 mg/kg b.wt) of carvacrol orally one week before DEN administration and up to 16 weeks	—	Cell proliferation	Carvacrol attenuates hepatocellular carcinoma by inhibiting cell proliferation and tumor metastases	Subramaniyan et al. (2014), India
	Post-treatment: (15 mg/kg b.wt) of carvacrol orally for 6 weeks after administration of DEN for 10 weeks		Tumor markers		
			Mast cell density		
			PCNA		
			MMP-2 and -9		
			AgNORs		
DMH-induced colon carcinogenesis in male Wistar rats who received subcutaneous injections of DMH (20 mg/kg b.wt) in the right thigh, once a week for the first 4 weeks of the experiment (four injections)	20, 40 or 80 mg/kg every day from the day of the carcinogen treatment till the end of the 16th week	Weight gain	Incidence of tumors	Carvacrol has antiproliferative, anticarcinogenic and chemopreventive potential and its effects were better observed at a dose of 40 mg/kg b.wt	Sivaranjani et al. (2016), India
		Growth rate	Growth of neoplastic polyps		
		GPx, GR, GSH, SOD, CAT			
C57BL/6 mice induced with DEN hepatocellular carcinoma injected intraperitoneally at a dose of 100 mg/kg	—	Intrastromal and peritumor lymphocytes	Tumor growth	The direct regulation relationship between DAPK1 and PPP2R2A may be the biological mechanism of tumorigenesis and progression of hepatocellular carcinoma	Li et al. (2019), China
	Intragastrically	DAPK1	Tumor cells		
	20 weeks		Mitotic phase		
			PPP2R2A		
DMBA-induced breast cancer in female Holztman mice in a single administration of DMBA by oral gavage at a dose of 80 mg /kg body weight	50, 100 and 200 mg/kg/day	Tumor latency	Number of tumors	Carvacrol had an antitumor effect on breast cancer *in vivo* and it is likely that this effect may be due to its antioxidant activity	Rojas-Armas et al. (2020), Peru
	Oral gavage		75% in the frequency of tumors		
	14 weeks		67% in the incidence of tumors		
			Average volume		

**TABLE 2:** Detailed description of the studies that used thymol, included in the systematic review.

Model	Concentration/Incubation time	Experimental methods for testing IC50 values	Results/targets			Conclusion	Authors (Year), Country
			Increase	Decrease	IC_50_		
Monoterpene thymol							
*In vitro* studies							
HepG2	150–900 μmol	–	Cytotoxic effects	DNA damage level	–	HepG2 cells were slightly more sensitive to the effects	Horváthová et al. (2006), Slovakia
Caco-2	24 of incubation						
K-562	200, 400, 600, 800, 1,000 μM	Trypan blue exclusion	Cytotoxic effects	DNA damage level	500 μM–24 h	Thymol has cytotoxic, antioxidant effects and has a protective action against DNA damage	Horvathova et al. (2007), Slovakia
	24 or 48 h of incubation						
P-815	0.004–0.5% v/v	MTT assay	Cytotoxic effects	–	0.015% v/v–48 h	Thymol is cytotoxic	Jaafari et al. (2007), Morocco
	48 h of incubation						
HepG2	150–1,000 μM	Trypan blue exclusion	Cytotoxic effects	Cell proliferation	HepG2 - 400 μM–24 h	Thymol has antiproliferative and protective effects	Slamenová et al. (2007), Slovakia
			Resistance to harmful DNA effects (antioxidant properties)				
Caco-2	24 of incubation				Caco-2 - 700 μM–24 h		
HeLa	15, 30.5, 61, 122, 244 ng/mL	–	Cytotoxic effects	Cell survival	–	Thymol has strong antitumor activity against the HeLa cell line	Abed, (2011), Iraq
Hep	72 h of incubation						
MG63	100, 200, 400, 600 μmol/L	–	Cytotoxic effects	Cell viability	–	Thymol showed antitumor activity in MG63 cells, moreover, its apoptotic effect is related to the pronounced antioxidant activity	Chang et al. (2011), China
	24 h of incubation		Apoptosis induction				
			Generation of ROS				
HL-60	5, 25, 50, 75, 100 μM	–	Cytotoxic effects	Cell viability	–	Apoptosis induced by thymol in HL-60 cells involves the dependent and independent pathways of caspase	Deb et al. (2011), India
	24 h of incubation		Apoptosis induction	Cells in phases G0/G1, S and G2/M			
			Cells in sub phase G0/G1 generation of ROS	Cell cycle stop in phase G0/G1 Bcl-2			
			Caspase-9, -8 and -3				
DBTRG-05MG	200, 300, 400, 500, 600, 800 μM	–	Cytotoxic effects	Cell viability	–	Thymol induces cell death in human glioblastoma cells	Hsu et al. (2011), China
			Apoptosis induction and necrosis				
	24 h of incubation						
HepG2	20–200 μg/mL	CellTiter-Blue^®^ cell viability assay	Cytotoxic effects	Membrane damage	60.01 μg/mL–24 h	Thymol exhibits antioxidant activities and anti-cancer effects on cells	Özkan and Erdogan, (2011), Turkey
			Antiproliferative effects				
	24 h of incubation						
P-815	0.05–1.25 μM	MTT assay	Cytotoxic effects	Cell cycle stop in phase G0/G1	P-815–0.15 μM–48 h	Thymol showed relevant cytotoxic effects in all tested strains	Jaafari et al. (2012), Morocco
CEM	48 h of incubation				CEM - 0.31 μM–48 h		
K-562					K-562–0.44 μM–48 h		
MCF-7					MCF-7 - 0.48 μM–48 h		
MCF-7_gem_					MCF-7_gem_ -		
H1299	10–2,000 μM	CellTiter-Blue^®^ cell viability assay	Cytotoxic effects	Membrane and DNA damage	497 μM–24 h	Thymol exhibited a cytotoxic and antioxidant effect	Ozkan and Erdogan, (2012), Turkey
	24 and 48 of incubation		MDA		266 μM–48 h		
			8-OHdG				
B16-F10	75, 150, 300, 600, 1,200 μM	Trypan blue and MTT assay	Cytotoxic effects	Cell viability	400 μM	Thymol showed antitumor effect with moderate cytotoxicity	Satooka and Kubo, (2012), United States
	24 h of incubation		Generation of ROS				
				Density of melanoma cells			
HepG2	1.56–50 μg/mL	Trypan blue assay	Cytotoxicity only for B16-F10 cells	–	HepG2 - > 25 μg/mL	Thymol showed cytotoxicity to B16-F10 cells	Ferraz et al. (2013), Brazil
K-562	72 h of incubation		Apoptosis induction in HepG2 cells		K-562–72 h		
B16-F10			Induction of caspase-3-dependent apoptotic cell death in HepG cells		B16-F10–18.23 μg/mL–72 h		
PC-3	10, 30.50, 70, 100 μg/mL	MTT assay	Cytotoxic effects	Cell viability	PC-3 - 18 μg/mL–48 h	Thymol exhibited cytotoxicity and induced apoptosis	Pathania et al. (2013), India
MDA-MB 231			Apoptosis induction	Cell proliferation	MDA-MB 231–15 μg/mL–48 h		
A549	48 h of incubation		DNA fraction sub G0	PI3K/AKT/mTOR	A549–52 μg/mL–48 h		
MCF-7			TNF-R1		MCF-7 - 10 μg/mL–48 h		
HL-60			Bax	Bcl-2	HL-60–45 μg/mL–48 h		
			Caspase-8 and 9				
Caco-2	100–2,500 μM	–	–	–	–	The cells exposed to thymol remained unchanged and did not produce any cytotoxic, apoptotic or necrotic effects at any of the tested concentrations	Llana-Ruiz-Cabello et al. (2014), Spain
	24 and 48 h of incubation						
A549	1–1.000 μM	SRB assay	Cytotoxic effects	–	A549–0.187 ± 0.061 mΜ–72 h	Thymol exhibited more effective cytotoxicity against cells (Hep3B), while cells (A549) were less sensitive to treatment and cells (HepG2) were more resistant	Fitsiou et al. (2016), Greece
HepG2	72 h of incubation		Antiproliferative effects		HepG2 - 0.390 ± 0.01 mΜ–72 h		
Hep3B					Hep3B- 0.181 ± 0.016 mΜ–72 h		
AGS	100, 200, 400 μM	–	Cytotoxic effects	Cell viability	–	Thymol has potent anticancer effects on gastric cancer cells	Kang et al. (2016), Republic of Korea
			Apoptosis induction				
			Sub-G1 phase	Cell growth			
	6, 12, 24 h of incubation		Generation of ROS				
			Bax	MMP			
			Caspase-8, -7 and -9				
C6	0.1, 0.3, 1, 3, 10,	–	–	Cell viability	–	Thymol is a potential candidate for the treatment of malignant gliomas	Lee et al. (2016), Republic of Korea
	30, 100, 200 µM			Cell migration			
	24 h of incubation			p-ERK1/2			
				MMP-2 and -9			
A549	0–250 μM	–	Cytotoxic effects	Cell viability	–	Thymol has cytotoxic and antioxidant activity and its cytotoxic effect was greater than that of carvacrol	Coccimiglio et al. (2016), Canada
	24 h of incubation						
HCT-116	100, 150, 200 μg/mL	–	Cytotoxic effects	Cell proliferation	–	Thymol can be used as a potent drug against colon cancer due to its lower toxicity	Chauhan et al. (2018), Republic of Korea
	24 h of incubation		Apoptosis induction	Clonogenic potential			
			Generation of ROS				
			Caspase-3				
			p-JNK				
			Cyt C				
HepG2	0.06, 0.11, 0.22, 0.45, 0.90 μg/μL	MTT assay	–	Cell viability	289 mg/L–24 h	Thymol has therapeutic potential in tumor cells without adverse effects on healthy cells	Elshafie et al. (2017), Italy
	24 h of incubation			Hepatocarcinoma cells			
T24	25, 50, 100, 150 μM	MTT assay	Cytotoxic effects	Cell viability	T24–90.1 ± 7.6 μM–24 h	Thymol can be used as a promising anticancer agent against bladder cancer	Li et al. (2017), China
SW780	24 h of incubation or 100 μM –		Apoptosis induction	Cell cycle stop in phase G2/M	SW780–108.6 ± 11.3 μM–24 h		
			p21	Cyclin A and B1			
J82	6, 12, 24, 36 h of incubation		Caspase-3 and -9	CDK2	J82–130.5 ± 10.8 μM–24 h		
			p-JNK				
			p-p38				
			MAPK	PI3K/Akt			
			Generation of ROS				
PC-3	100, 300, 500, 700, 900 μM	–	Cytotoxic effects	Cell viability	–	Thymol was cytotoxic to PC-3 cells	Yeh et al. (2017), China
	24 h of incubation		Induction of cell death				
Cal7	200–800 µM	Cell Titer 96 ^®^ Aqueous non-Radioactive cell Proliferation assay	Cytotoxic effects	Cell viability	350 μM–500 μM	Thymol had cytotoxic, antiproliferative and antitumor effects	De La Chapa et al. (2018), United States
SCC4	48 h of incubation						
SCC9							
HeLa							
H460							
MDA-231							
PC-3							
AGS	10, 20, 30, 50, 100, 200, 400, 600 µM	CellTiter-Glo Luminescent cell viability assay	Apoptotic effects	Cell viability	75.63 ± 4.01 µM–24 h	Thymol has cytotoxic, apoptotic, genotoxic and dose-dependent ROS-generating effects	Günes-Bayir et al. (2018), Turkey
	24 h of incubation		Necrosis	Bcl-2			
			Bax				
			Caspase-3 and -9				
			Generation of ROS	GSH levels			
			Genotoxic effect				
MCF-7	10, 15, 30, 50, 80, 100, 200 μg/mL	MTT assay	Cytotoxic effects	Bcl-2	MDA-MB 231–56 μg/mL–24 h	Thymol has antiproliferative effects	Jamali et al. (2018), Iran
MDA-MB 231	24 h of incubation		Antiproliferative effect	Interruption of cell cycle progression in the S phase	MCF-7 - 47 μg/mL–24 h		
			Apoptosis induction				
			Caspase-3				
			Bax				
			Generation of ROS				
			Sub-G1 phase				
MCF-7	5, 10, 20, 30, 40, 50, 75, 100 g/mL	MTT assay	Cytotoxic effects	Number of cancer cells	54 μg/mL - 48 h	Thymol can induce the process of apoptosis in MCF-7 and, therefore, can be considered an anticancer agent	Seresht et al. (2019), Iran
	48 and 72 h of incubation		p53	Cell cycle arrest induction	62 μg/mL - 72 h		
			p21				
HT-29	62.5, 125, 250, 500, 750, 1,000 ppm	Trypan Blue exclusion assay	Cytotoxic effects	–	152.1 ± 18.0 ppm–24 h	Thymol induces cytotoxicity and provides genoprotective effects	Thapa et al. (2019), United Kingdom
	24 h of incubation		Genoprotective effects				
MDA-MB 231	100, 200, 400, 600, 800 µM	MTT assay	Cytotoxic effects	–	MDA-MB 231–208.36 μM–72 h;	Thymol has apoptotic and antiproliferative properties and can serve as a potential therapeutic agent	Elbe et al. (2020), Turkey
PC-3	24, 48 and 72 h of incubation		Antiproliferative effect		PC-3 - 711 μM–24 h, 601 μM–48 h and 552 μM–72 h;		
DU 145			Apoptosis induction		DU 145–799 μM–24 h, 721 μM–48 h and 448 μM–72 h		
KLN 205					KLN 205–421 μM–48 h and 229.68 μM–72 h		
SKOV-3	100, 200, 400, 600 μM	MTT assay	Apoptosis induction	Cell viability	316.08 μM–24 h	Thymol was cytotoxic to the ovarian cancer cell line and it was more potent than carvacrol	Elbe et al. (2020), Turkey
	24 and 48 h of incubation				258.38 μM–48 h		
HCT116	10, 20, 40, 80, 120 μg/mL	CCK-8 Kit	Apoptosis induction	Proliferative capacity	LoVo - 41.46 μg/mL - 48 h	Thymol treatment reduced the proliferative capacity of cells and suppressed cell migration and invasion	Zeng et al. (2020), China
					HCT116–46.74 μg/mL - 48 h		
LoVo	24, 48 and 72 h of incubation		Bax	Cell migration and invasion			
			Caspase-3 and PARP	Cell cycle stop			
			Cells in phase G0/G1	Bcl-2			
				Cells in S and G2/M phases			
AGS	0–600 μM	CellTiter-Glo Luminescent cell viability assay	Cytotoxic effects	Cell viability	75.63 ± 4.01 μM–24 h	Thymol has cytotoxic and antioxidant effects in gastric adenocarcinoma	Günes-Bayir et al. (2020), Turkey
	24 h of incubation		Generation of ROS	GSH levels			
			Apoptosis induction				
			Bax	Bcl-2			
			Caspase-3 and -9				
			DNA damage				
A549	25–200 μg/mL	MTT assay	Antiproliferative effect	Cell viability	745 μM–24 h	Thymol can act as a safe and potent therapeutic agent to treat non-small cell lung cancer	Balan et al. (2021), India
	12 and 24 h of incubation		Apoptosis induction	MMP			
			DNA damage	Bcl-2			
			Generation of ROS				
			Caspase-3 and -9				
			Bax	SOD			
			Cells in phase G0/G1				
			TBARBS				
			CARBONIL				
KG1	25, 50, 100 μM	–	Cell death	Cell viability	–	KG1 cells treated with 50 µM thymol were more sensitive compared to the other two lines. At 100 μM, thymol induced complete cell death of KG1 and HL60 cells, while about 50% of K562 cells resisted cell death after 48 h of treatmentl	Bouhtit et al. (2021), Belgium
K-562	24 and 48 h of incubation						
HL-60							

Model	Concentration	Results/Targets		Conclusion	Authors (Year), country
		Increase	Decrease		
*In vivo studies*					
Female athymic nude rats were injected subcutaneously in the right flank with 3 × 10^6^ Cal27 or HeLa cells in 0.1 mL of sterile PBS	4.3 mM thymol (32 μg diluted in 50 μl sterile saline with a final concentration of 0.25% DMSO)	Apoptotic cells	Tumor volume reduction	Thymol had cytotoxic, antiproliferative and antitumor effects	De La Chapa et al. (2018), United States
			Proliferative cells		
Xenograft model: BALB/c male nude mice were injected subcutaneously with HCT116 cells (1 × 10^7^ cells in 0.2 mL of PBS) on the back	Xenograft model and lung metastasis model: Intraperitoneal injection for 30 days with thymol at 75 mg/kg 1x on alternate days or thymol at 150 mg/kg 1x on alternate days	Necrotic lesions	Tumor growth and metastasis	Thymol inhibits the growth and metastasis of colorectal cancer *in vivo* by suppressing Wnt/β-catenin signaling and the EMT program	Zeng et al. (2020), China
			Average number of tumor nodules on the surface of the lungs		
		Bax	Ki-67 expression level		
Lung metastasis model: HCT116 cells (1 × 10^6^) were intravenously injected into the tail vein of each mouse			Cell proliferation		
			Bcl-2		
			Wnt/β-catenin signaling pathway		
		Caderina-E	Vimentina		
			Cyclin D1		
			C-myc		
			Survivin		
Male Wistar rats injected with DMH (40 mg/kg intraperitoneally, twice a week) for 16 consecutive weeks	20 mg/kg/day, orally, for 16 weeks	Final body weight	Mortality	Thymol administration had promising preclinical protective efficacy by promoting inhibition of oxidative stress, inflammation and induction of apoptosis	Hassan et al. (2021), Egypt
		Weight gain	Incidence of ACF		
		Growth rate	Serum CEA levels		
		NRF2	Serum levels of CA19-9		
			Caspase-3		
			TNF-α		
		GST, GSH, SOD, CAT	NF-κB		
			IL-6		
			Tissue content of MDA (colon lipid peroxidation)		

Abbreviations: 5RP7, Mouse embryonic fibroblast with transformation of H-ras oncogenes; 8-OHdG, 8-hydroxy-2′-deoxyguanosine; A375, Melanoma (skin) cancer cell line; A549, Lung Carcinoma Cell Line; ACF, Aberrant crypt foci; AFP, Alpha-fetoprotein serum; AFU, Alpha l-fucosidase; AgNORs, Proteins Associated with the Argyrophilic Nucleolar Organizing Region; AGS, Human gastric carcinoma cell line; ALP, Alkaline Phosphatase; ALT, Alanine transaminase; AST, Aspartate transaminase; AXL, Tyrosine Kinase Receptor; B[a]P, 3.4 benzopurene; B16-F10, Mouse melanoma cells; BT-474, Breast ductal carcinoma; BT-483, Breast ductal carcinoma; C6, Glioma cell line; Caco-2, Cell line derived from human colon carcinoma; CA 19–9, Tumor markers carbohydrate antigen 19–9; Cal27, Cell line of the squamous cell carcinoma of the tongue; CAT, Catalase; CEA, Carcinoembryonic antigen; CCK-8, Cell Counting Kit-8; CCND1, Gene encoding the cyclin D1 protein; CDK4 or 6, Cyclin-dependent kinases; cGT, Glutamyl transpeptidase Range; CyT C, Cytochrome C; c-Myc, Proto-oncogene; CO25, Mouse muscle cell line; COX-2, Cyclooxygenase; DAPK1, Protein kinase 1 associated with death; DBTRG-05MG, Human Glioblastoma Cells; DEN, Diethylnitrosamine; DMH, 1,2-dimethylhydrazine; DMBA, 7,12-dimethylbenz[a]anthracene; DMSO, Dimethylsulfoxide; DNA, Deoxyribonucleic acid; DU 145, Human Prostate Cancer Cell Line; EC50, Half of the maximum effective concentration; EMF, Acute T Lymphoblastoid Leukemia; EMT, Epithelial-mesenchymal transition; ERK 1/2, Kinase 1/2 regulated by extracellular signal; ERO, Reactive Oxygen Species; GGT, Gamma-Glutamyltransferase; GPx, Glutathione Peroxidase; GR, Glutathione reductase; GSH, Reduced Glutathione; H1299, Parental and Drug Resistant Human Lung Cancer Cell Line; H460, Non-small cell lung cancer cell line; HCT116, Colorectal adenocarcinoma cell line; HeLa, Human Cervical Cancer Cell Line; Hep, Human Laryngeal Squamous Cell Carcinoma; Hep3Β, Human Hepatocellular Carcinoma Cell Line; HepG2, Human Hepatocellular Carcinoma Cell Line; HL-60, Human Acute Promyelocytic Leukemia Cell Line; HT-29, Colorectal adenocarcinoma cell line; IC50, Half of the maximum inhibitory concentration; IL-6, Interleukin-6; J82, Bladder Cancer Cell Line; Jagged-1, Jagged Canonical Notch Ligand 1; JAR, Human Choriocarcinoma Cell Line; JEG3, Human Choriocarcinoma Cell Line; Jurkat, Lymphocytes derived from T-cell lymphoma; KG1 and K-562, Human Myelogenous Leukemia Cell Line; Kelly, Neuroblastoma cell line; Ki-67, Antigen, biomarker; KLN 205, Non-small cell lung cancer; LDH, Lactate dehydrogenase; LoVo, Colorectal Adenocarcinoma Cell Line; MAPK, Protein kinase activated by mitogen; MTT, Methyl Tetrazolium Test; MTS, Tetrazolium salt reduction; MCF-7, Human breast cancer cell line; MCF-7gem, Gemcitabine-resistant human breast adenocarcinoma; MDA, Malondialdehyde; MDA-MB 231, Human metastatic breast adenocarcinoma cell line; MDA-MB 453, Human metastatic breast adenocarcinoma cell line; MDPK, Myotonic dystrophy protein kinase; MG63, Human Osteosarcoma Cell Line; MMP, Potential of the mitochondrial membrane; MMP-2 or 9, Metalloproteinase-2 or 9 of the matrix; N2a, Rat neuroblastoma cell line; NDEA, N-nitrosodiethylamine; Notch-1, Signaling path; NSCLC, Non-small cell lung cancer; OC2, Human oral cancer cells; OSCC, Human oral squamous cell carcinoma; p21, WAF1 encoding gene; p38, Mitogen-activated protein kinases; p53, tumor protein; P-815, Murine Mastocytoma Cell Line; p-AKT, Phospho-protein kinase B; PBS, Sterile phosphate buffered saline; PC-3, Human Prostate Cancer Cell Line; PCNA, Proliferating Cell Nuclear Antigen; PI3K/AKT/mTOR, Phosphoinositide-3-kinase/Akt/mammalian target; PI3K/Akt, Phosphoinositide-3-kinase-Akt; p-JNK, Fosto-c-Jun N-terminal kinase; p-p38, Phospho-p38; PPP2R2A, Serine/threonine-protein phosphatase 2A; p-STAT3, Phospho-signal transducer and transcription activator; SRB, Sulforhodamine B; SCC-25, Human squamous cell carcinoma cell line; SCC4 and SCC9, Human oral squamous cell carcinoma cell line; SH-SY5Y, Neuroblastoma cell line; SiHa, Human Cervical Cancer Cell Line; SKOV-3, Ovarian cancer cell line; SOD, Superoxide dismutase; SW780, Bladder cancer cell line; T24, Bladder Cancer Cell Line; TAC, Total antioxidant capacity; TBARS, Thiobarbituric Acid Reactive Substances; TCA-8113, Human tongue squamous cell carcinoma cell line; TNF-α, Tumor Necrosis Factor-Alpha; TNFR1, Tumor necrosis factor 1 receptor; TOS, Total oxidant status; TRPM7, Subfamily M of the cation channel of the potential transient receptor Member 7; U87, Human glioblastoma cell line; VEGF, Vascular endothelial growth factor; XXT, 2.3‐bis(2‐methoxy‐4‐nitro‐5‐sulfophenyl)‐2H‐tetrazolium-5-carboxanilide inner salt.

### Description of *In Vitro* Studies with Carvacrol and Thymol

#### Carcinomas

Carvacrol (500 and 1,000 μM) was able to inhibit the viability and proliferation of lung cancer cells (A549 cell line), in addition to inducing early apoptotic characteristics (Koparal and Zeytinoglu, 2003) and reducing the viability of the A549, H460 (Jung et al., 2018) and H1299 cells lines, the latter being resistant to epirubicin (Ozkan and Erdogan, 2012). These effects occurred mainly through the inhibition of tyrosine kinase receptor (AXL) expression and an increase in malondialdehyde (MDA) and 8-hydroxy-2′-deoxyguanosine levels (8-OHdG) (Ozkan and Erdogan, 2012; Jung et al., 2018).

In relation to hepatocarcinomas (HepG2 cell line), carvacrol exhibited anticancer effects, provoking cell death and antiproliferative effects in a concentration-dependent manner (Özkan and Erdogan, 2011; Melusova et al., 2014). The inhibition of cell proliferation and apoptosis induction occurred via the mitochondria-mediated pathway, accompanied by caspase-3 activation and Bcl-2 inhibition (Yin et al., 2012). The via extracellular signal-regulated kinases (ERK) protein, and mitogen-activated protein kinases (p38) apoptotic pathways may also be involved (Yin et al., 2012). Similarly, Melušová et al. (2014) demonstrated a marked apoptotic effect of carvacrol at a concentration of 650 μM after 24 h of incubation, and an accumulation of cells in the G1 phase, together with a reduction of cells in the S phase, slowing cell cycle/mitosis and provoking cell death.

Colorectal cancer (Caco-2 cell line) also exhibited reduced cell viability and a significant increase of early apoptotic cells after carvacrol incubation (115 μM) (Llana-Ruiz-Cabello et al., 2014). There was also inhibition of HCT116, LoVo and HT-29 cells proliferation (Fan et al., 2015; Pakdemirli et al., 2020). Carvacrol also promoted a decrease in Bcl-2, metalloproteinase-2 and -9 (MMP-2 and MMP-9), p-ERK, p-Akt, cyclin B1 levels and an increase in p-JNK, Bax levels, resulting in cell cycle arrest at the G2/M phase (Fan et al., 2015).

In respect of breast cancer, treatment with carvacrol decreases MDA-MB231 (Jamali et al., 2018; Li et al., 2021) and MCF-7 cells line viability (Al-Fatlawi and Ahmad, 2014; Jamali et al., 2018; Tayarani-Najaran et al., 2019; Li et al., 2021). At 200 μM, the MDA-MB-231 cell line was the most sensitive and MCF-7 was the least sensitive, indicating that the effectiveness of carvacrol may vary according to the types of breast cancer cell. In addition, the TRPM7 pathway is one of the suggested pharmacological mechanisms of action (Li et al., 2021). Carvacrol was more cytotoxic compared to thymol (Jamali et al., 2018), α-thujone, 4-terpineol, 1,8-cineol, bornyl acetate and camphor (Tayarani-Najaran et al., 2019). Tayarani-Najaran et al. (2019) also reported an apoptotic effect marked by an increased level of Bax protein, and cleaved both poly [ADP-ribose] polymerase 1 (PARP-1) and caspase-3. The antiproliferative activity of carvacrol was 1.2 times higher against MDA-MB231 cells compared to U87 cells (Baranauskaite et al., 2017). MDA-MB 231 cell proliferation slowed after treatment with carvacrol, accompanied by apoptosis induction with increased levels of Bax, decreased mitochondrial membrane potential, cytochrome C release, caspase activation, PARP cleavage, increased sub-phase G0/G1 of the cell cycle and a reduced number of cells in the S phase (Arunasree, 2010). The viability of MCF-7 cells was reduced after carvacrol treatment (200 μmol/L), with a significant increase in the number of early and late apoptotic cells, accompanied by a negative regulation of Bcl2 and positive regulation of Bax protein. An accumulation of cells in the G0/G1 phase was observed, along with a reduction of cells in the S and G2 phases, mainly through the reduced expressions of CDK4, CDK6, retinoblastoma protein (pRB), cyclin D and phosphoinositide-3-kinase-Akt (PI3K/p-AKT) (Mari et al., 2020).

It was also observed that the administration of carvacrol provoked cytotoxic and apoptotic effects on HeLa and SiHa cervical cell lines (Mehdi et al., 2011). In fact, Potočnjak et al. (2018) demonstrated that the cytotoxicity exhibited by carvacrol against HeLa cells occurred through the suppression of the cell cycle and induction of apoptosis, the latter accompanied by an increase in caspase-9, PARP cleavage, and activation of ERK, increasing the expression of phospho-ERK1/2. In SiHa cells, the reduction in viability and apoptosis induction occurred through p53 activation and Bax, caspase-3, -6, -9 expression, along, with negative regulation of Bcl-2 gene (Abbas and Al-Fatlawi, 2018). Furthermore, another study demonstrated that carvacrol and thymol were cytotoxic against ovarian cancer (SKOV-3 cell line) exhibiting apoptotic and antiproliferative properties (Elbe et al., 2020).

Carvacrol also induced cytotoxicity and apoptosis (via caspase-3 and reactive oxygen species—ROS) of human oral squamous cell carcinoma (OC2 cell line) in a concentration-dependent manner (Liang et al., 2013). In tongue cancer (Tca-8113, SCC-25 cell lines), Dai et al. (2016) reported that carvacrol effectively inhibited cell proliferation through the negative regulation of CCND1 and CDK4 expression, and the positive regulation of p21 expression, resulting in a significant decrease of cells in the S phase, in addition to inhibiting the migration and invasion abilities of Tca-8113 cells via phospho-focal adhesion kinase (p-FAK), p-catenin, ZEB1 and MMP-2 and -9 reduction. Apoptosis was marked by a reduction of anti-apoptotic Bcl-2 proteins expression and an increase of proapoptotic Bax proteins levels (Dai et al., 2016).

In a prostate cancer cell line (DU 145), carvacrol showed a significant reduction of cell viability and proliferation in a concentration and time dependent manner, marked by a cell cycle arrest, resulting in the accumulation of cells in the G0/G1 phase, and apoptosis, related to the increased activity of caspase-3, production of ROS and loss of mitochondrial membrane potential (Khan et al., 2017). PC-3 cells also exhibited cytotoxicity and decreased cell viability in a concentration dependent manner after carvacrol treatment (Horng et al., 2017). A blockade of TRPM7 channels, reduced expression of MMP-2 and F-actin, was also observed, together with the inhibition of PI3K/Akt and MAPK (Mitogen-activated protein kinases) signaling pathways was also observed (Luo et al., 2016). Similarly, Heidarian and Keloushadi (2019) reported that this monoterpene acts through the negative regulation of pERK1/2, pSTAT3 and pAKT expression, suggesting that inhibition of interleukin-6 (IL-6) signaling pathways can be a promising target for prostate cancer treatment. The induction of PC-3 cells apoptosis was mostly through the intrinsic pathway, associated with the production of ROS and mediated by the increase expression of caspase-3, -8 and -9 and Bcl-2/Bax. There was also a G0/G1 phase arrest of cell cycle, together with a considerable decrease of cells in the S and G2/M phase (Khan et al., 2019). Similarly, Tayarani-Najaran et al. (2019) demonstrated decreased cell viability in a concentration dependent manner and also marked apoptosis (mitochondrial pathway), accompanied by cleavage of PARP-1 and caspase-3 and an increased Bax protein level.

Günes-Bayir et al. (2018), Günes-Bayir et al. (2018), and Maryam et al. (2015) reported cytotoxic effects of carvacrol on gastric cancer (AGS cell line) significantly reducing cell viability in a manner dependent on concentration. There was also an induction of apoptosis with a reduction of Bcl-2 protein levels, and an increase in Bax, caspase-3 and -9 protein levels, besides the production of ROS. In their most recent study, Günes-Bayir et al. (2020) also identified the cytotoxic effects of thymol on AGS cell viability, in addition to inducing apoptosis, by increasing ROS, Bax, Caspase-3, -9 levels and reducing Bcl-2 and GSH levels.

Regarding human choriocarcinoma (JAR and JEG3 cell lines), carvacrol was able to inhibit proliferation and induce cell cycle arrest. The results showed that carvacrol reduced cell proliferation and provoked apoptosis mediated by mitochondrial membrane potential depolarization, increased mitochondrial calcium and activation of Bax and Cytochrome C expression. In addition, treatment with this monoterpene promoted an accumulation of cells in the sub-G1 phase, indicating that changes in intracellular calcium and ROS generation are related to the antiproliferative effects observed. Additionally, there was a marked phosphorylation of ERK1/2 and also inhibition of the PI3K/AKT signaling pathway, indicating that carvacrol regulates signaling pathways by inhibiting MAPK and PI3K (Lim et al., 2019).

In murine B16-F10 and A375 melanoma cell lines, carvacrol reduced cell viability and induced cytotoxicity (Satooka and Kubo, 2012; Ferraz et al., 2013; Govindaraju and Arulselvi, 2018). The antiproliferative effect was confirmed by Govindaraju and Arulselvi (2018), who reported marked cell cycle arrest, attested by the accumulation of G1 phase cells, a reduction in the number of G2/M cells and apoptosis through the mitochondria-mediated pathway and PARP cleavage/activation, together with a reduced expression of the anti-apoptotic protein Bcl-2.

Similarly, the administration of thymol to lung cancer cells promoted a reduction in cell viability in the A549, H460 and H1299 cell lines (Pathania et al., 2013; Coccimiglio et al., 2016; De La Chapa et al., 2018; Balan et al., 2021). Likewise, the cytotoxic effect of thymol on A549 cells was higher than carvacrol cytotoxicity (Coccimiglio et al., 2016). Thymol also promoted cytotoxicity and apoptosis of KLN 205 cells with an IC_50_ of 421 and 229.68 μM in 48 and 72 h, respectively (Elbe et al., 2020). In liver carcinoma cells (HepG2), thymol exhibited antioxidant activity at lower (<IC_50_ = 60.01 μg/mL) concentrations and antitumor effects (apoptosis and inhibition of cell proliferation) at higher concentrations (>IC_50_ = 60.01 μg/mL) (Özkan and Erdogan, 2011). Elshafie et al. (2017) reported HepG2 cell death, decreased cell viability and a selective action of thymol against these tumor cells.

Thymol also showed concentration-dependent cytotoxic effects and reduced the proliferation of Caco-2 cells (Horváthová et al., 2006). In contrast, Llana-Ruiz-Cabello et al. (2014) reported that Caco-2 cells exposed to thymol did not exhibit any cytotoxic, apoptotic or necrotic effects in any of the tested concentrations. HCT-116 and HT-29 cells, after thymol administration, displayed a cell number reduction, cell apoptosis by disrupting mitochondrial membrane potential and ROS production (Chauhan et al., 2018; Thapa et al., 2019). These effects may have been caused by the positive regulation of the caspase-3, PARP-1, p-JNK and Cytochrome C expression (Chauhan et al., 2018).

Breast cancer cells (MDA-MB 231 cell line) also exhibited a reduction in cell viability after thymol treatment (Pathania et al., 2013; De La Chapa et al., 2018; Elbe et al., 2020). The inhibition of cell proliferation and apoptosis on MDA-MB231 and MCF-7 cell lines occurred via the mitochondrial pathway and induction of oxidative damage to DNA through Bax/Bcl-2 modulation, decreased levels of procaspase-8, -9, -3, increased levels of cleaved caspase-3 and ROS, and also cell cycle arrest at S-phase (Jamali et al., 2018). According to the results found by Seresht et al. (2019), thymol produced cytotoxic effects and reduced the number of MCF-7 cells, suggesting that this monoterpene induces cell cycle arrest, probably due to p21 overexpression. Thymol also promoted a marked antitumor effect on cervical cancer (HeLa cell line), through cytotoxic effects on the concentration of 30.5 ng/mL (Abed, 2011). De La Chapa et al. (2018) also reported decreased viability of HeLa cells and induction of apoptosis by PARP cleavage, suggesting that the anticancer effect of thymol is caused by mitochondrial dysfunction and subsequent apoptosis.

The administration of thymol to bladder cancer (T24, SW780, J82 cell lines) provoked inhibition of cell proliferation and decreased the cell viability in a concentration and time dependent manner, along with marked cell cycle arrest in the G2/M phase and induction of apoptosis through the intrinsic pathway, together with the activation of caspase-3 and -9, JNK and p38, release of cytochrome C, negative regulation of Bcl-2 family proteins and production of ROS. In addition, a considerable decrease in the expression of cyclin A, B1 and CDK2, as well as an increase in the expression of p21 were observed after treatment with thymol, suggesting that its antitumor effect occurs by inhibiting the PI3K/Akt signaling pathway, via MAPKs, and generation of ROS (Li et al., 2017). In human laryngeal squamous cell carcinomas (Hep), thymol showed a pronounced reduction of cell proliferation and also apoptosis, at a concentration of 30.5 ng/mL. According to De La Chapa et al. (2018) thymol exhibited cytotoxicity and decreased cell viability in a concentration dependent manner on Cal27, SCC4 and SCC9 cell lines. However, this cytotoxicity was reversed by the N-acetyl-cysteine (NAC) antioxidant addition, providing evidence that the anticancer mechanism of action of thymol involves mitochondrial dysfunction, and generation of ROS, culminating in apoptosis (De La Chapa et al., 2018). Thymol also caused a decrease in cell viability of prostate cancer (PC-3 cell line), and provoked cytotoxic effects (Pathania et al., 2013; Yeh et al., 2017; De La Chapa et al., 2018). PC-3 cells demonstrated greater sensitivity to treatment with thymol compared to DU145 cells. In addition, the induction of apoptosis in both cell lines occurred in a concentration-dependent manner (Elbe et al., 2020). Similarly, thymol suppressed the viability of melanoma (B16-F10 cell line), also in a concentration-dependent manner, by reducing the cell number and provoking cytotoxic effects. These effects seem to be related to the oxidative damage observed after the increase of ROS levels (Satooka and Kubo, 2012).

#### Central Nervous System Cancers

Human glioblastoma cells (DBTRG-05MG) showed reduced viability in a concentration-dependent manner when treated with carvacrol (200–600 μM), induced apoptosis and necrosis by ROS production and caspase-3 activity (Liang and Lu, 2012). In a rat neuroblastoma (N2a cell line), treatment with carvacrol (200–400 mg/L) exhibited cytotoxic and antiproliferative effects, along with antioxidant activity (Aydın et al., 2014). Kelly and SH-SY5Y neuroblastoma cells also exhibited a reduced proliferation rate after exposure to carvacrol (Kocal and Pakdemirli, 2020). In glioblastoma (cell line U87), carvacrol induced apoptosis by increasing the levels of caspase-3 cleavage, moreover, its antitumor mechanism of action seems to be related to the inhibition of PI3K/Akt signaling pathways, activation of mitogen/protein kinase by extracellular signals (via MAPK/ERK) and decreased levels of MMP-2 protein (Chen et al., 2015).

Regarding thymol, treatment at concentrations of 100 and 200 µM induced a significant reduction in cell viability and inhibited the migration of glioma cells (C6 cell line) through phosphorylation of PKCα and ERK1/2, that resulted in decreased expression of MMP-9 and MMP-2 (Lee et al., 2016). In addition, in DBTRG-05MG cells, thymol exhibited a cytotoxic effect in a concentration-dependent manner, by reducing cell viability and inducing apoptosis. The 400–600 μM range of concentrations promoted cell necrosis and the 800 μM concentration killed all cultivated cells (Hsu et al., 2011).

#### Sarcomas

Treatment with carvacrol in leiomyosarcoma cells exhibited antiproliferative effects in a concentration dependent manner and also inhibition of cell growth (Karkabounas et al., 2006). In addition, carvacrol showed a greater cytotoxicity compared to thymol against murine mast cell cells (P-815 cell line) (Jaafari et al., 2007; Jaafari et al., 2012), with accumulation of cells in the S phase (Jaafari et al., 2012).

In relation to thymol, there were concentration-dependent cytotoxic effects and interruption of the cell cycle progression in the G0/G1 phase in P-815 cells (Jaafari et al., 2012). In human osteosarcoma cells (MG63 cell line), thymol reduced cell viability, induced cytotoxic effects and apoptosis, which occurred in a concentration-dependent manner. Additionally, there was an increase in the production of ROS and cell death (Chang et al., 2011).

#### Leukemias

Carvacrol showed cytotoxic effects against human myeloid leukemia cells (K-562 cell line) (Horvathova et al., 2007; Jaafari et al., 2012) and against T-cell acute lymphoblastic leukemia (CEM cell line) (Jaafari et al., 2012). Carvacrol was more cytotoxic than thymol, inducing accumulation of cells in the S phase (Jaafari et al., 2012). It was shown that carvacrol produced cytotoxic effects and reduced cell viability in human acute promyelocytic leukemia (HL-60 cell line) and lymphocytes derived from T-cell lymphoma (Jurkat cell line). Treatment with carvacrol (100 μM) showed early and late apoptotic cells accompanied by a reduction of mitochondrial membrane potential levels, suggesting that apoptosis was mediated by the mitochondrial pathway, with a significant increase of Bax pro-apoptotic proteins, decreased expression of the anti-apoptotic proteins Bcl2 and an increased caspase-3 protein level (Bhakkiyalakshmi et al., 2016).

Analyzing the effects of thymol on K-562 and CEM cells, Jaafari et al. (2012) revealed that the latter was more sensitive to thymol effects, resulting in the accumulation of cells in the G0/G1 phase. In addition, treatment with thymol also reduced HL-60 cell viability, exhibiting cytotoxicity with concentrations above 50 μM (Deb et al., 2011). Cell cycle arrest was observed in the G0/G1 phase, with decreased Bcl-2 protein levels and interruption of mitochondrial homeostasis; increased ROS production, mitochondrial production of H_2_O_2_, and Bax protein levels; and activation of caspase-8, -9, -3 and PARP (Deb et al., 2011). Thus, inhibition of the PI3K/Akt/mTOR signaling pathway may be a possible mechanism involved behind the effects of thymol on HL-60 cells (Pathania et al., 2013).

It was observed by Bouhtit *et al.* (2021) that at a concentration of 300 µM of carvacrol the KG1 cell lines were more sensitive compared to the HL60 cell line, and at 400 µM the K-562 cell line showed resistance after 48 h of treatment. Regarding thymol (50 µM), the KG1 cell line was also more sensitive when compared to the other two and at the 100 µM dose, thymol was able to induce complete cell death in the KG1 and HL60 cell lines (Bouhtit et al., 2021).

#### Transformed Cell Lines

When using mouse myoblast cells (CO25 cell line) transformed with human N-RAS oncogene, Zeytinoglu et al. (2003) showed that the concentrations of 1, 5, and 10 μg/mL of carvacrol provoked cytotoxic effects. The same effects were also observed for 5RP7 and CO25 cells transformed by H-RAS and N-RAS oncogenes, respectively, as well as apoptotic morphological changes in both cell lines. However, the fragmentation of internucleosomal DNA and the initial apoptotic determinants were observed only in the cell line 5RP7 cell line. In addition, H-RAS-transformed 5RP7 cells were more sensitive to carvacrol than N-RAS-transformed CO25 cells (Akalin and Incesu, 2011).

Based on these data, we compiled the IC_50_ (μM) values determined 24 h after the incubation of the studied cells with carvacrol or thymol. It was possible to verify that, in general, carvacrol (336.7 ± 35.0, *n* = 21) is more potent than thymol (527.1 ± 146.6, *n* = 11), with difference between means of 103.8 (±106.9). The lowest IC_50_ values for carvacrol were against prostate carcinoma (PC-3 IC_50_ = 46.71 μM, Khan et al., 2019; DU 145 IC_50_ = 84.39 μM, Khan et al., 2017) and gastric carcinoma (AGS IC_50_ = 82.57 μM, Günes-Bayir et al., 2018), whereas thymol appears to be more selective for gastric cancer carcinoma (AGS, IC_50_ = 75.63 μM, Günes-Bayir et al., 2018) as seen in Supplementary Table S2.

### Description of *In Vivo* Studies with Carvacrol and Thymol

Anticarcinogenic effects were observed after treatment with carvacrol in Wistar rats, depicted by a reduction in the incidence of tumors, increased survival rate, and a reduced carcinogenic potency of the substance in inducing malignant tumors (Karkabounas et al., 2006). The pre- and post-treatment with carvacrol in animals with liver cancer induced by diethylnitrosamine (DEN) revealed a decrease in the number of nodules, a final body weight increase and a reduction in liver weight. In fact, carvacrol pre-treatment caused the disappearance of most tumoral foci and nodules, characterized by few neoplastic cells, suggesting a chemopreventive effect. In contrast, post-treatment with carvacrol demonstrated the presence of small persistent nodules, loss of cellular architecture and a lower tendency to spread through the intrahepatic veins. Moreover, carvacrol was able to increase the levels of superoxide dismutase (SOD), catalase (CAT), glutathione peroxidase (GPx), glutathione reductase (GR) and glutathione (GSH), along with a reduction of lipid peroxides and the enzymes AST, ALT, ALP, LDH and γGT in the serum (Jayakumar et al., 2012).

Similarly, Subramaniyan et al. (2014) also evaluated the effect of carvacrol pre- and post-treatment on a DEN-induced hepatocarcinogenesis rat model and observed a stability in tumor marker levels, a reduced mast cell density and inhibition of cell proliferation. Furthermore, supplementation with carvacrol significantly restored the activities of liver microsomal xenobiotic metabolizing enzymes to normal, with a reduced expression of proliferative nuclear cell antigen (PCNA), MMP-2 and -9, and thereby prevented the local spread of carcinogenic cells, showing an antimetastatic effect (Subramaniyan et al., 2014). Hence, in a rat model of hepatocellular carcinoma induced by diethylnitrosamine (DEN), carvacrol treatment promoted DNA fragmentation indicating its potential as an apoptotic agent. In addition, carvacrol showed a reduction in serum levels of alpha-fetoprotein (AFP), alpha l-fucosidase (AFU), vascular endothelial growth factor (VEGF) and decreased expression of the gamma glutamyl transferase (GGT) gene (Ahmed et al., 2013).

Carvacrol supplementation significantly improved the weight gain and growth rate of animals with colon cancer induced by 1,2-dimethylhydrazine (DMH), exhibiting a lower incidence of tumors and pre-neoplastic lesions, along with a reduction in oxidative stress damage (higher levels of GSH, GPx, GR, SOD and CAT), suggesting that carvacrol presents chemopreventive effects (Sivaranjani et al., 2016).

Li et al. (2019) showed that tumor growth in mice with DEN-induced hepatocarcinoma and treated with carvacrol was limited, revealing tumor cell reduction, rare mitotic figures, normal arrangement of cells, few microvessels, a central necrotic area on tumor tissue and a reduction of intrastromal and peritumor lymphocytes. Likewise, there was an increased expression of the death-associated protein kinase 1 (DAPK1) and decreased expression of serine/threonine-protein phosphatase 2A (PPP2R2A) in tumor tissues (Li et al., 2019). More recently, Rojas-Armas et al. (2020) showed a better effect of carvacrol at a dose of 100 mg/kg/day compared to the other doses tested (50 and 200 mg/kg/day) in female Holztman rats with breast cancer induced by 7,12-dimethylbenzanthracene (DMBA), showing a reduction of 4 (of 16) tumors, in addition to a 75% reduction in the frequency of tumors, a 67% reduction in incidence, an increase in tumor latency and a reduction in the average tumor volume and cumulative tumor volume (Rojas-Armas et al., 2020).

De La Chapa et al. (2018), after treating female athymic nude mice injected with tongue squamous cell carcinoma (Cal27 cell line) and cervical cancer (HeLa cell line), reported a significant inhibition of tumor growth and volume, besides a significant reduction in the number of proliferative cells, with thymol increasing the quantity of apoptotic cells. In a later study, Zeng et al. (2020) established two *in vivo* models to investigate the effect of thymol on cancer progression. For the colorectal cancer model, HCT116 xenograft was injected (i.p.) into BALB/c mice, which after 7–10 days (when the tumors grew to approximately 100 mm^3^) were treated with thymol (75 or 150 mg/kg every other day). A significant reduction in cancer growth, a greater number of necrotic lesions and a lower level of Ki-67 expression were observed, which reflects cell proliferation. As for the lung metastasis model, HCT116 cells were injected into the tail vein of each mouse and then received treatment with thymol (75 or 150 mg/kg every other day). After 6 weeks, they found that the average number of tumor nodules on the lung surface of the two treatment groups was significantly lower, revealing an anti-metastatic effect, probably due to the inhibition of the Wnt/β-catenin signaling pathway (Zeng et al., 2020).

**FIGURE 2:**
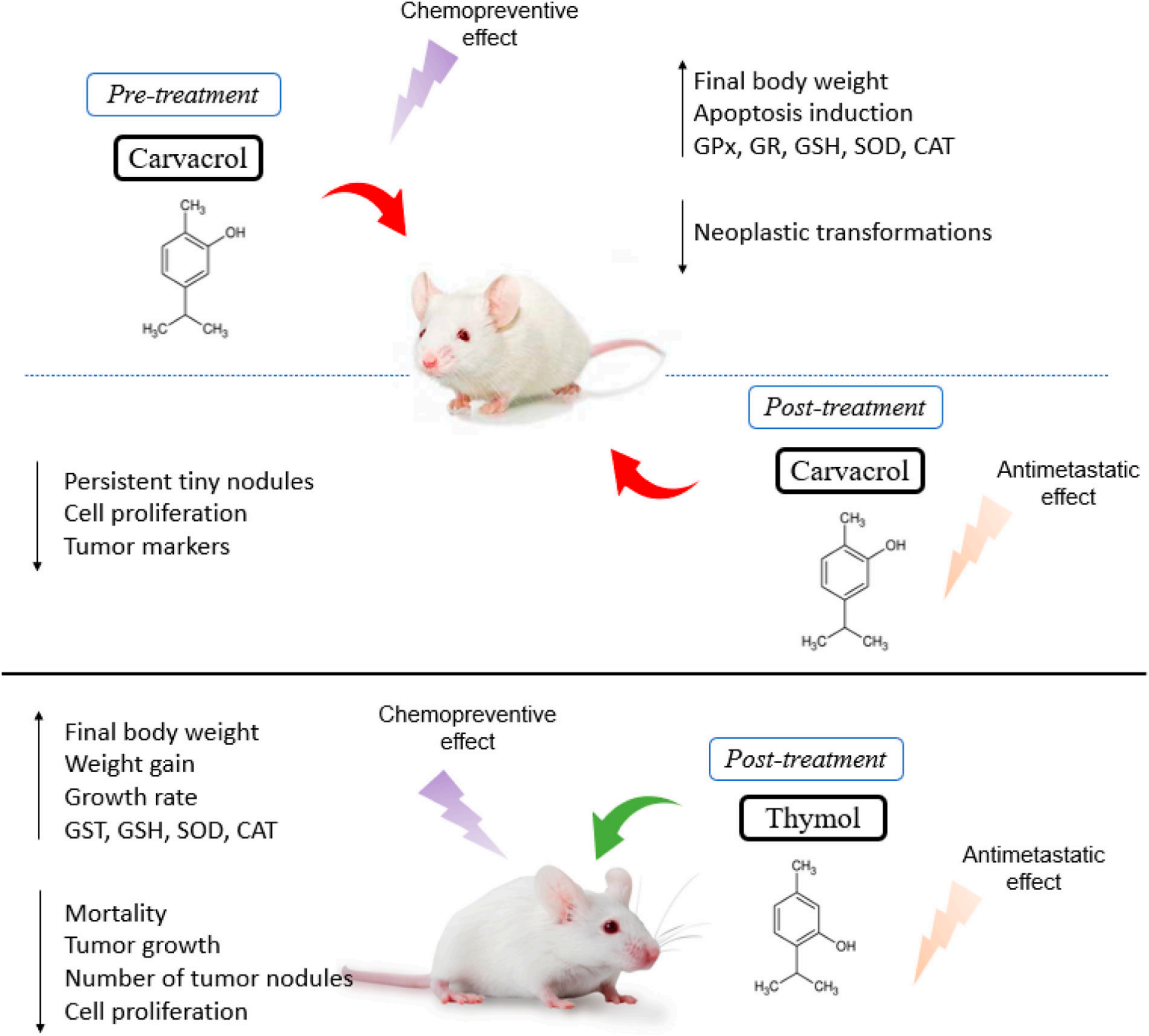
Summary of main effects observed in *in vivo* studies.

It was revealed in the study by Hassan et al. (2021) that the administration of thymol (20 mg/kg/day, p. o.) in male Wistar rats provided promising protective activity against colon cancer by significantly reducing elevated serum levels of colon-related tumor markers, carbohydrate antigen 19-9 (CA 19-9) and carcinoembryonic antigen (CEA), as well as the apoptotic marker, caspase-3 compared to the colon cancer group. In addition, it promoted the reduction of oxidative stress by increasing the enzymatic antioxidants SOD, CAT, GSH and GST, inhibiting inflammation by decreasing TNF-α, NF-κB and IL-6 (Hassan et al., 2021). Figure 2 shows a summary of the main effects observed in the *in vivo* studies.

### Risk of Bias and Quality of Included Studies

**FIGURE 3:**
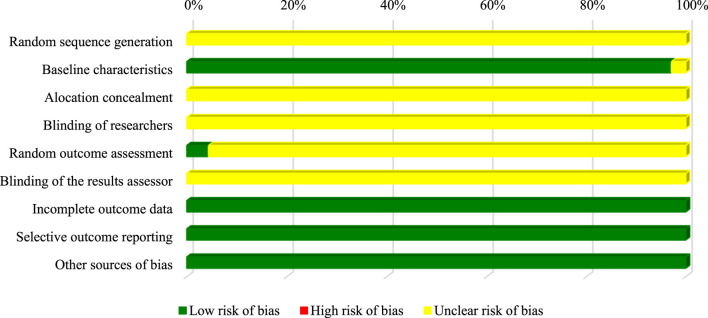
Assessment of *in vitro* studies risk of bias.

Almost all studies did not present enough data to allow the judgment of the domains related to the generation of the random sequence, concealment of the allocation, blinding of the researchers and evaluators of the results. Only 4.3% (*n* = 3) of the studies recorded reported randomness when taking photos of selected areas regarding apoptotic activity assays. However, 97% of the studies applied the same conditions (temperature and incubation time, and purity, stability) for *in vitro* assays, ensuring a low risk of bias (Figure 3).

**FIGURE 4:**
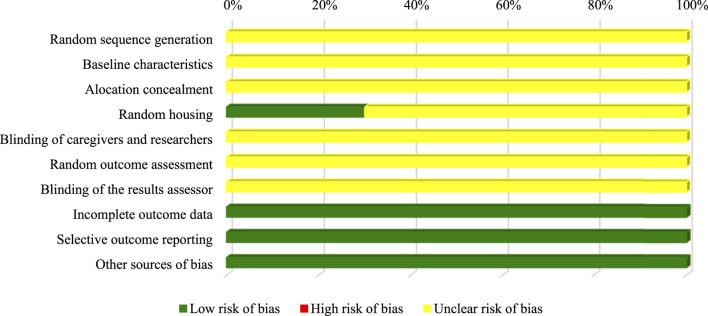
Assessment of *in vivo* studies risk of bias.

Similarly, most of the SYRCLE tool domains for animal studies (random sequence generation; baseline characteristics; allocation concealment; blinding of caregivers and researchers; random evaluation of results; blinding of the results evaluator) were classified as uncertain due to the lack of information in the articles. In contrast, 30% (*n* = 3) record random allocation of the animals in the study (Figure 4).

## Discussion

Through the scientific evidence compiled in this systematic review, it was possible to verify the preventive and therapeutic effects of carvacrol and thymol in cancer in addition to the antimetastatic activity that these compounds were able to exert due to their cytotoxic and antiproliferative effects. Most studies have demonstrated the effect of these compounds on carcinomas, as they are one of the most common types of cancer (Neville, 2011).

Medicinal plants and their bioactive compounds have been an important source of recent drug discoveries (Van Wyk and Wink, 2018). Our review identified a considerable number of reports (77 studies) published in the last two decades, with an increasing trend over recent years. In fact, phytochemicals have great pharmaceutical significance due to their diverse structures (with more than 100,000 being described so far) and their pharmacological properties (Srivastav et al., 2020), and have already made an important contribution to cancer treatments (Ashraf, 2020). Our review also showed that carvacrol and its thymol isomer are capable of restraining growth and combating different tumor strains *in vitro*.

In Asia, the continent with the largest number of publications on the subject, the use of traditional, popular medicine continues to grow (De Boer and Cotingting, 2014) due to the low costs, easy access, the frequent reduced side effects and toxicity, and their better biodegradable properties (Soković et al., 2013). This is often reinforced by difficulties in accessing health services and obtaining essential medicines (WHO, 2007-2017; Ozawa et al., 2019). Moreover, India and China have, historically, made important contributions to knowledge about medicinal plants, being responsible for some of the most ancient reports about this issue, that were written approximately 5,000 and 4,000 years ago, respectively (Wiart, 2007; Kelly, 2009).

**FIGURE 5:**
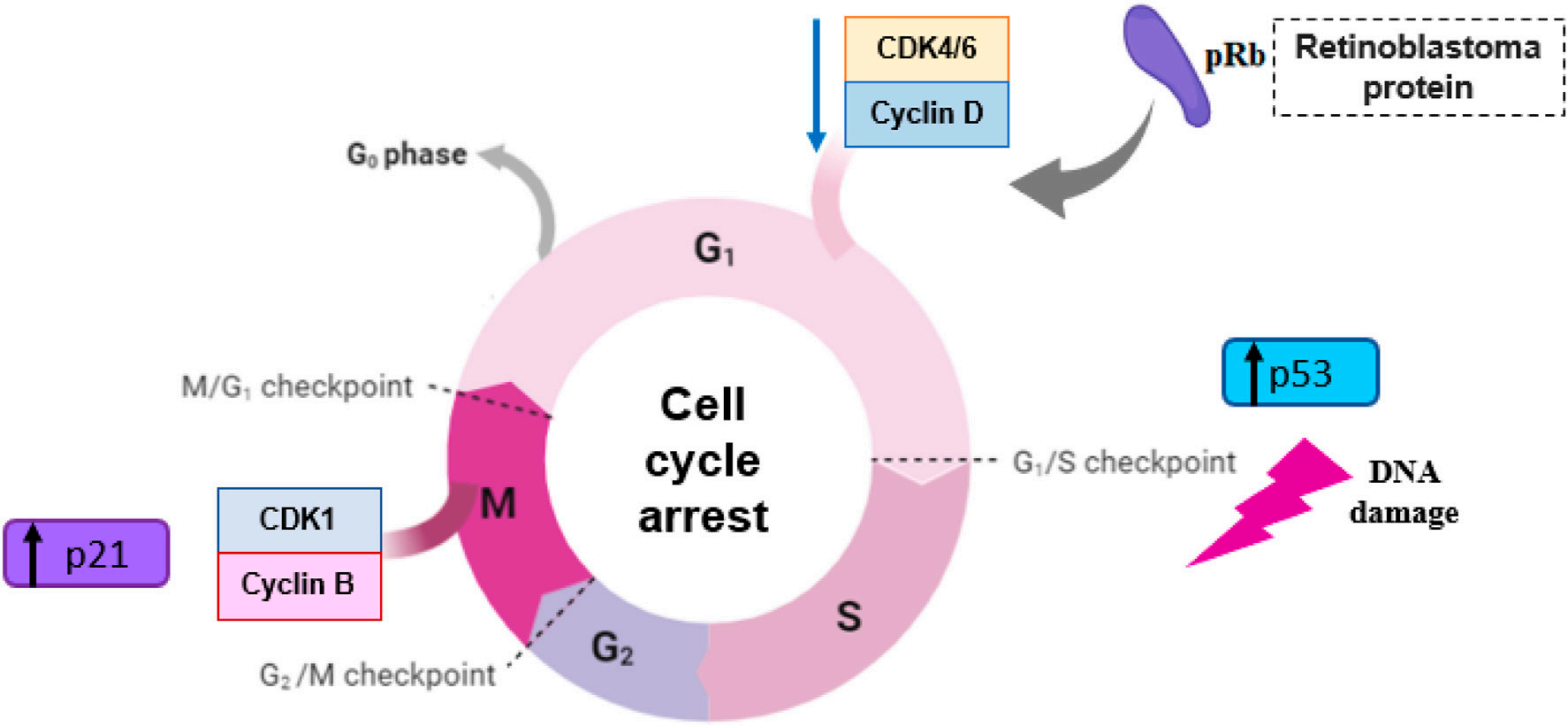
Cell cycle involvement in cancer treatment with carvacrol and thymol.

Neoplasia can be described as a disease of unchecked cell division, and its progression is related to abnormal activity of cell cycle regulators. The cell cycle consists of four discrete phases in which the cell increases in size and cellular content is duplicated (gap 1 or G1 phase), DNA is replicated (synthesis, or S phase), it prepares to divide (gap 2, or G2 phase), and then divides, creating two identical daughter cells (mitosis, or M phase). As a cell moves through each phase, stimulated by growth and transcription factors, it passes through several checkpoints, which ensure that mitosis occurs only when the cellular genome has been precisely replicated, avoiding mutations and generation of transformed cells (Hamilton and Infante, 2016; Ingham and Schwartz, 2017). The cell cycle is controlled mainly by cyclin-dependent kinases (CDKs) (Ingham and Schwartz, 2017) CDK4/6 are the kinases responsible for the inactivation/phosphorylation of retinoblastoma protein, at the G1/S phase transition checkpoint. In this review, it was noted that three *in vitro* studies (4.4%) reported that carvacrol decreased CDK4 protein expression in human tongue squamous cell carcinoma (Tca-8113 cells) (Dai et al., 2016) and in breast cancer (MCF-7 cells) (Mari et al., 2020; Li et al., 2021) and only one (1.4%) reported a decrease in CDK6 in MCF-7 cells (Mari et al., 2020). CDK inhibitor drugs are being used in some cancer types (Mari et al., 2020), such as acute myeloid leukemia (Lee and Zeidner, 2019) and breast cancer (Pernas et al., 2018). In addition, cell cycle arrest, reported in 14.7% (*n* = 10) of *in vitro* studies also represents a promising target of cancer treatment, since natural compounds can act as modulators, interrupting the cell cycle and, therefore, killing cancer cells (Bailon-Moscoso et al., 2017). The uncontrolled proliferation of cancer cells occurs due to their ability to prevent programmed cell death (apoptosis) (Olsson and Zhivotovsky, 2011; Dabrowska et al., 2016; Kim and Kim, 2018), that is responsible for eliminating aberrant proliferating cells or those with DNA damage/mutations (Dabrowska et al., 2016) (Figure 5). In this review, 55% (*n* = 38) of the studies reported induction of apoptosis after treatment with carvacrol or thymol in their *in vitro* studies and only 40% (*n* = 4) in *in vivo* studies.

The tumor suppressor TP53 gene, also called “the guardian of the genome”, is activated in response to stress signals (DNA damage) and can interrupt DNA replication and cell division through cell cycle arrest (G1/S checkpoint), in order to restore genetic integrity, thereby preventing genetically transformed cell proliferation (Harris and Levine, 2005; Belyi et al., 2010; Georgakilas et al., 2017). When repair is not possible, p53 causes programmed cell death, interacting with the Bcl-2 family of proteins, triggering genes involved in apoptosis, such as Bax (Gottlieb and Oren, 1998; Song et al., 2014). In addition, evidence indicates that when there is a deficiency of p53, the p21 gene can act as an oncogenic factor, causing the cell cycle to be interrupted (Georgakilas et al., 2017). Treatment with carvacrol increased p53 expression in breast cancer (Al-Fatlawi and Ahmad, 2014), cervical and liver cancer cells (Abbas and Al-Fatlawi, 2018). Meanwhile, administration of thymol increased p21expression in bladder cancer cells (Li et al., 2017) and increased expression of both p53 and p21 in breast cancer cells (Seresht et al., 2019). In fact, TP53 genetic alterations are commonly observed in clinical tumor samples, since most mutations lead to function loss, in which cells can escape the destruction and repair process, resulting in a malignant transformation through favorable natural selection (Olsson and Zhivotovsky, 2011).

**FIGURE 6:**
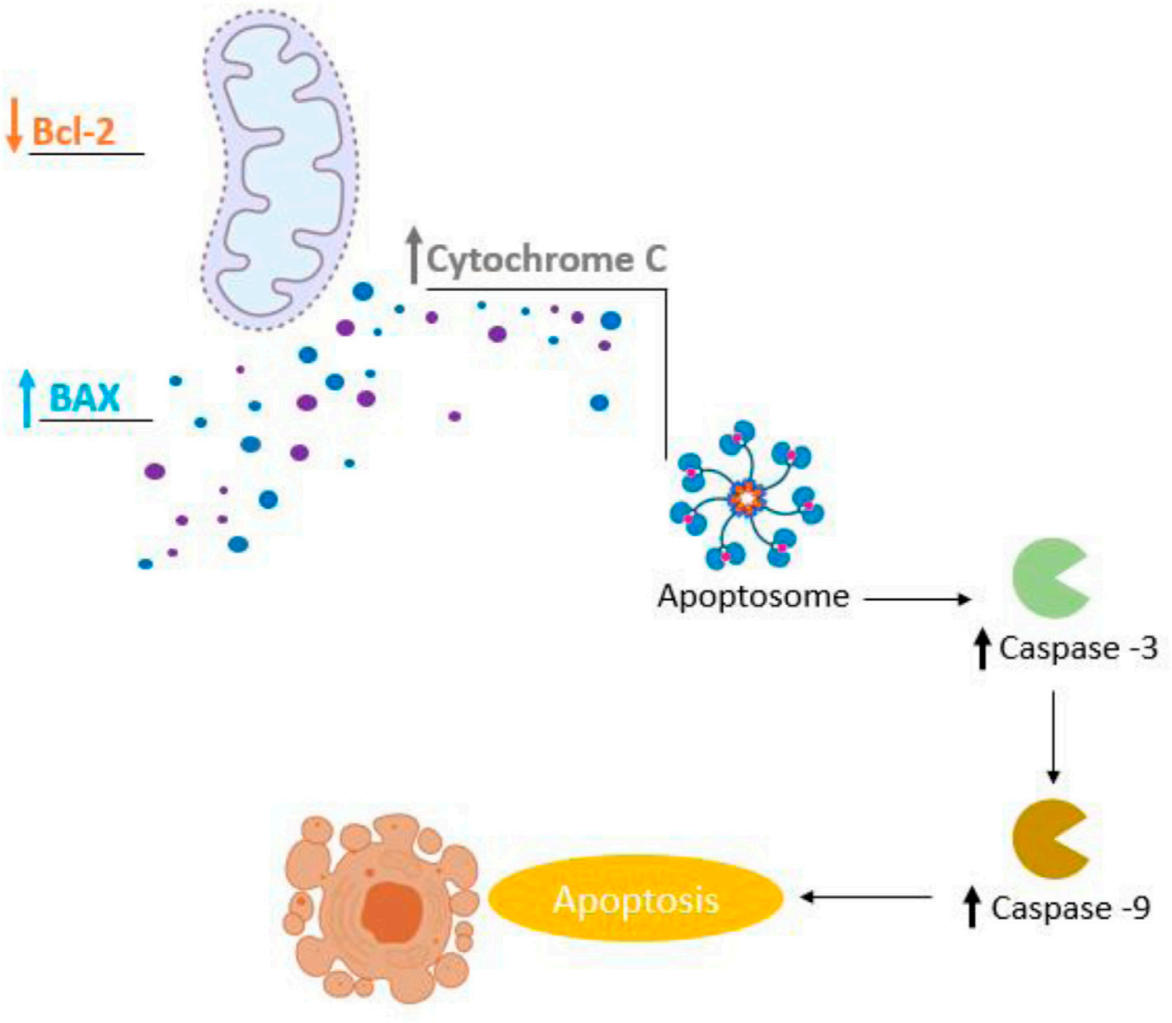
Involvement of apoptosis in carvacrol and thymol treatment in cancer.

During cancer, overexpression of anti-apoptotic genes and under expression of pro-apoptotic genes can lead to the failure of the programmed cell death mechanism (Olsson and Zhivotovsky, 2011; Zhang et al., 2000). The expression of proapoptotic proteins, such as Bax (Li et al., 2017; Basu and Haldar, 1998), is an important mechanism of tumor regression (Backus et al., 2002). It triggers apoptosis by forming pores within the outer mitochondrial membrane, releasing cytochrome C that activates proteases such as caspase-9 and -3, that dismantle and destroy the cell (apoptosis) (Olsson and Zhivotovsky, 2011). Our results showed that 24.6% of *in vitro* studies (*n* = 17) and 10% of *in vivo* studies showed an increase in Bax levels after treatment with carvacrol or thymol. Additionally, 26% (*n* = 18) of *in vitro* studies reported negative regulation of the anti-apoptotic protein Bcl-2 (Swanton et al., 1999; Kroemer et al., 1998). Caspases are central apoptosis regulators and executors, and thus attractive targets for the development of therapeutic strategies for cancer treatment (Ghavami et al., 2009; Hensley et al., 2013; Fiandalo and Kyprianou, 2012; Shalini et al., 2015). Hence, positive expression of caspases increases tumor sensitization to treatment (Hensley et al., 2013) (Figure 6). In this review, 33.3% (*n* = 23) of *in vitro* studies reported an increase of caspase activity, more specifically caspase-3 (Deb et al., 2011; Liang and Lu, 2012; Yin et al., 2012; Ferraz et al., 2013; Liang et al., 2013; Al-Fatlawi and Ahmad, 2014; Chen et al., 2015; Bhakkiyalakshmi et al., 2016; Khan et al., 2017; Li et al., 2017; Abbas and Al-Fatlawi, 2018; Chauhan et al., 2018; Günes-Bayir et al., 2018; Günes-Bayir et al., 2018; Tayarani-Najaran et al., 2019), -6 (Al-Fatlawi and Ahmad, 2014; Abbas and Al-Fatlawi, 2018), -7 (Kang et al., 2016), -8 (Deb et al., 2011; Pathania et al., 2013; Kang et al., 2016; Khan et al., 2019) and -9 (Deb et al., 2011; Pathania et al., 2013; Al-Fatlawi and Ahmad, 2014; Kang et al., 2016; Li et al., 2017; Abbas and Al-Fatlawi, 2018; Günes-Bayir et al., 2018; Günes-Bayir et al., 2018; Potočnjak et al., 2018; Khan et al., 2019). It is noteworthy that the positive regulation of caspase-3 propagates and amplifies the apoptosis signal, and its loss of expression promotes tumorigenesis (Fiandalo and Kyprianou, 2012). Shalini et al. (2015) reported that high caspase-3 expression caused apoptosis of tumor cells, and is significantly associated with better prognosis in patients with non-small cell lung cancer (Yoo et al., 2004) and hepatocellular carcinomas (Huang et al., 2010). However, there is very little knowledge about the role of caspases-6 and -7 during cancer (Ghavami et al., 2009). In this review, caspase-6 and -7 expression was reported in only three studies. Overexpression of caspase-8 has been reported in prostate cancer cells treated with carvacrol (Khan et al., 2019) and in studies that tested thymol on promyelocytic leukemia (Deb et al., 2011; Pathania et al., 2013) and gastric cancer (Kang et al., 2016). The literature reveals that the activation of caspase-8 plays an important role in the initiation phase of apoptosis (Soung et al., 2005), as well as in suppressing oncogenic transformation, confirmed by an increased susceptibility to spontaneous mutations in its absence (Olsson and Zhivotovsky, 2011). Krelin et al. (2018) reported that caspase-8 deficient cells exhibit resistance to death, facilitating tumorigenic transformation (Krelin et al., 2008). The expression of caspase-9 was the second most reported in this review, due to its crucial role in apoptosis initiation among various types of cancer (Kim et al., 2015). Previous studies have reported that caspase-9 regulates the apoptosis process of cancer cells through interactions with signaling molecules (Bou-Hanna et al., 2015; Thakor et al., 2017). Natural compounds can regulate caspase-9 expression, and, therefore, favor apoptosis in cancer (Kim et al., 2015). Pre-treatment with caspase inhibitors caused a significant reduction in cytotoxicity and attenuation of apoptosis induced by carvacrol, observed in prostate cancer cells (Khan et al., 2019).

**FIGURE 7:**
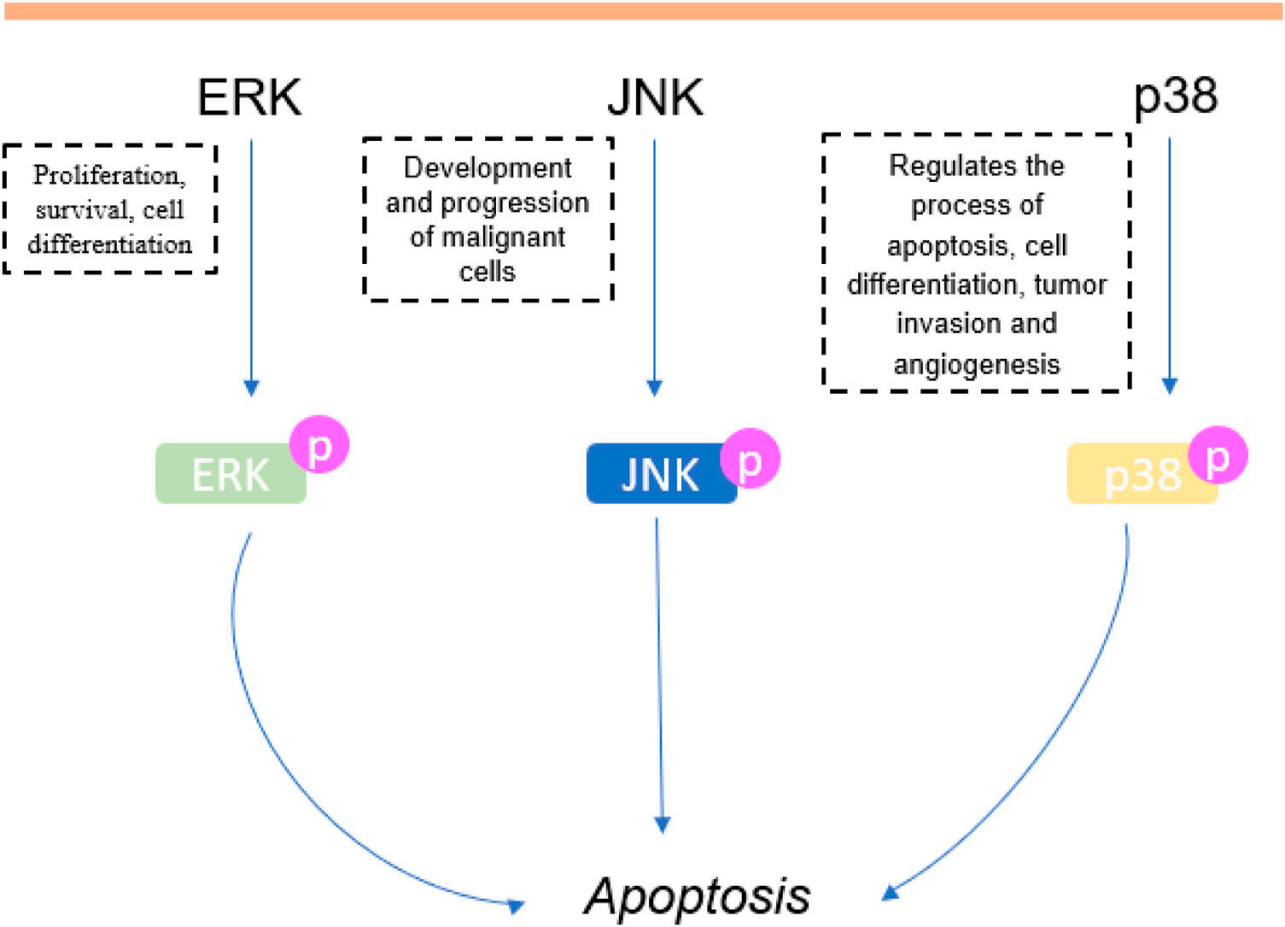
Involvement of the MAPK pathway in carvacrol and thymol treatment in cancer.

Another very important cell cycle regulator is MAPK. It controls cell growth, proliferation, differentiation and apoptosis, and represents one of the main signaling pathways involved in extracellular signals transduction (Binétruy et al., 2007; Kim and Choi, 2015; Li et al., 2018). In particular, JNK, ERK and p38 are the main proteins of MAPK pathways when approaching cancer. ERK is generally associated with cell proliferation, while JNK and p38 are closely related to the cell death process (Wagner and Nebreda, 2009). JNK is proven to be related to the development and progression of malignant cells (Wu et al., 2019). In this review, 5.7% (*n* = 4) of the studies that tested carvacrol or thymol showed that they were able to increase *in vitro* JNK phosphorylation in colon cancer (Fan et al., 2015; Chauhan et al., 2018), choriocarcinoma (Lim et al., 2019) and bladder cancer (Li et al., 2017). Studies showed that phosphorylation/activation of p38, MAPK and JNK contributes to cancer cell apoptosis (Liu et al., 2014; Wang et al., 2014) and that the p38 regulates apoptosis process, cycle growth progression and cell differentiation (Zarubin and Han, 2005; Krens et al., 2006). P38 can also directly affect tumor invasion and angiogenesis (Wagner and Nebreda, 2009). Herein, 4.3% (*n* = 3) of the studies *in vitro* induced phosphorylation of p38 in hepatocarcinoma cells (Yin et al., 2012), cancer bladder cells (Li et al., 2017) and choriocarcinoma cell (Lim et al., 2019). In fact, Li et al., (2017) suggested that the activation of JNK and p38 were pivotal to the cytotoxicity exhibited by thymol against cancer cells. In addition, the level of phosphorylated ERK decreased in hepatocarcinoma, colon cancer, choriocarcinoma and prostate cancer cells, after treatment with carvacrol, and p-ERK1/2 levels decreased after thymol treatment in glioma cells (Yin et al., 2012; Fan et al., 2015; Heidarian and Keloushadi, 2019; Lim et al., 2019). This corroborated the findings of Wang et al. (2018) who reported that patients with tumors with low p-ERK (activated form) had a higher survival rate (Low and Zhang, 2016) (Figure 7).

The PI3K/AKT/mTOR signaling pathway is also studied in cancer since it regulates cell proliferation, growth, metabolism and motility (Song et al., 2019; O'Donnell et al., 2018). Its inhibition induces a pronounced anticancer activity (Li et al., 2016). In addition, some inhibitors of this signaling pathway have already been approved by the Food and Drug Administration for cancer treatment (Alzahrani, 2019). PI3k belongs to a family of lipid kinases, involved in extracellular signals transduction and cell growth promotion (Wullschleger et al., 2006; Alzahrani, 2019). AKT, also known as protein kinase B (PKB), is an oncogenic protein that regulates cell survival, proliferation, growth, apoptosis and glycogen metabolism (Alzahrani, 2019; Song et al., 2019). Excessive activation of mTOR (mammalian target of rapamycin), a serine/threonine kinase, is associated with the activation of hypoxia inducible factor (HIF) that regulates angiogenesis and tumor growth (Semenza, 2003). Its inhibition was identified by 2.8% (*n* = 2) of *in vitro* studies using thymol in human promyelocytic leukemia cell lines (Pathania et al., 2013) and bladder cancer cells (Li et al., 2017), and by 7.2% (*n* = 5) of *in vitro* studies that used carvacrol in colon cancer (Fan et al., 2015), glioblastoma (Chen et al., 2015), prostate cancer (Luo et al., 2016), choriocarcinoma (Lim et al., 2019) and breast cancer cells (Mari et al., 2020).

The mechanism of action reported in *in vivo* studies involves slightly more complex processes. Starting with Ahmed et al. (2013), the only authors of this review who reported in *in vivo* models a reduction in serum levels of AFP, AFU, VEGF and reduced GGT gene expression after treatment with carvacrol. The literature reveals that AFP, besides being one of the most useful biomarkers for the detection of hepatocellular carcinoma, also serves to monitor the response to anticancer therapy (high levels indicate tumor progression) (Bei and Mizejewski, 2011; Wong et al., 2015; Wang and Wang, 2018). On the other hand, AFU indicates clinical prognosis of several malignant tumors and helps to diagnose primary hepatocarcinoma (Giardina et al., 1992), colorectal cancer (Ayude et al., 2000), ovarian cancer (Abdel-Aleem et al., 1996), and was recently identified as an effective new biomarker for squamous cell carcinoma of the early esophagus (Yu et al., 2019). In addition, high levels of GGT influence proliferation and apoptosis and contribute to tumor progression (Koss and Greengard, 1982; Pompella et al., 2006; Zhang et al., 2006) serving as a biomarker in various types of cancer (Paolicchi et al., 1996; Whitfield, 2001; Pompella et al., 2006). The increased expression of VEGF during cancer, along with other pro-angiogenic factors, is responsible for new vascularization, representing a strategic point in the treatment (Collins and Hurwitz, 2005; Riaz et al., 2015; Siveen et al., 2017). The decrease in serum levels of these biomarkers in animals treated with carvacrol may prove to be an important antitumor characteristic that deserves attention and further studies to elucidate this mechanism (Ahmed et al., 2013). Together with VEGF, other enzymes collaborate for neoplastic invasion, such as mitochondrial membrane potential. They are matrix degradation enzymes (such as MMP), responsible for the degradation of the main constituents of basement membrane and extracellular matrix, facilitating the invasion of tumor cells and favoring the spread of cancer cells and metastases (Nagase et al., 2006; Shuman Moss et al., 2012; Jabłońska-Trypuć et al., 2016). High MMP-2 and -9 levels were associated with esophageal carcinomas (Koyama et al., 1990) and breast (Alrehaili et al., 2020), oral (Lin et al., 2004), bladder (Fouad et al., 2019), skin (Fundyler et al., 2004), larynx (Liu et al., 2005) cancer. In this systematic review, only one *in vivo* study (10%) reported a decrease in the levels of MMP-2 and MMP-9 in liver cancer after treatment with carvacrol (Subramaniyan et al., 2014), the majority 8.6% (*n* = 6) were *in vitro* studies. It is important to note that carvacrol prevented metastasis *in vivo*, demonstrated by tumors less likely to spread through intrahepatic veins (Jayakumar et al., 2012) and preventing local spread of cancer cells by suppressing the expression of MMP-2 and MMP-9 proteins (Subramaniyan et al., 2014), and thymol decreased the number of lung metastatic lesions *in vivo*, suppressing the Wnt/β-catenin signaling pathway (Zeng et al., 2020). However, there is almost nothing about this topic documented in the literature, and further studies are required to address this outcome.

Considering reactive oxygen species (ROS), these are a group of molecules that contain reduced forms of oxygen with short life and that are more energetically reactive than molecular oxygen (Srinivas et al., 2019). The generation of ROS inside the cell contributes to the antitumor process in order to induce DNA damage and slow the progression of the cell cycle, preventing cells with DNA damage (cancer cells) from continuing with cell division (Allawzi et al., 2019; Srinivas et al., 2019). *In vitro*, carvacrol and thymol increased the generation of reactive oxygen species in 24.63% (*n* = 17) of the studies, a fact that is also observed in chemotherapeutics such as doxorubicin (Conklin, 2004), cisplatin (Marullo et al., 2013) and bleomycin (Allawzi et al., 2019), which also increase ROS levels. Regarding *in vivo* studies, the pretreatment with carvacrol in colon cancer (Sivaranjani et al., 2016) and hepatocellular carcinoma (Jayakumar et al., 2012) increased the levels of enzymatic antioxidants such as GPx, SOD, CAT, GR, and GSH, revealing a chemopreventive effect of carvacrol and prevention of cell proliferation (Jayakumar et al., 2012; Sivaranjani et al., 2016). Similarly, thymol had a promising protective efficacy, revealing a chemopreventive effect against colon cancer observed by the increase in GST, GSH, SOD and CAT levels, in addition to inhibiting oxidative stress (Hassan et al., 2021). However, these results should be interpreted with caution, as low levels of ROS can be beneficial in preventing the development of cancer cells, since they can promote cancer (Prasad et al., 2017). In this sense, anticancer therapies can follow two paths; using compounds that prevent the formation of ROS, and thereby preventing carcinogenesis; or using compounds that have as their action mechanism the increase of ROS, promoting oxidative stress within the tumor (de Sá Junior et al., 2017). Further studies are needed to clarify this issue.

Post-treatment with carvacrol also promoted an increase in the expression of the DAPK1 gene and a decrease in the enzyme PPP2R2A (Li et al., 2019). DAPK1 is a serine/threonine kinase, a tumor suppressor protein that promotes apoptosis (Agodi et al., 2015; Zhai et al., 2019), while PPP2R2A, a regulatory subunit of protein phosphatase 2A (PP2A), controls the pathway of AKT signaling associated with tumor growth (Wang et al., 2016; Zeng et al., 2016). In fact, both may be involved in a possible antitumor mechanism of carvacrol, but it is still not very clear, requiring further studies (Li et al., 2019).

Through *in vitro* studies, we found that carvacrol proves to be more potent than thymol, and appears to have a greater cytotoxic effect for some cell lines, such as carcinomas (prostate and stomach) (Khan et al., 2017; Günes-Bayir et al., 2018; Khan et al., 2019). Thymol seems to act more against gastric cancer carcinoma (AGS, IC_50_ = 75.63 μM, Günes-Bayir et al., 2018). In fact, two recent studies published by Sisto et al. (2020) and Sisto et al. (2021) demonstrated the potential of these monoterpenes in the control of gastric carcinoma by reducing the viability of AGC cells.

In *in vivo* studies, on the other hand, the effect of carvacrol has been predominantly studied against hepatocarcinoma, with few studies for breast and colon cancer. In a way, it can be seen that the studies in this area do not seem to evolve toward a specific target, with low complementarity of the screenings carried out *in vitro* for the studies developed with experimental animals. This is a great barrier advances in this area of knowledge and helps to explain the discrepancy in the number of studies published *in vitro* (*n* = 69) and *in vivo* (*n* = 10) over the last two decades, as well as the absence of clinical trials. Thus, it is necessary to consider the studies already carried out for these compounds before conducting new primary studies on this topic, in order to evolve the research stages to the next level of drug development: *in vivo* studies and clinical trials.

As for security, some studies have suggested that normal cells tolerate exposure to carvacrol (Koparal and Zeytinoglu, 2003; Yin et al., 2012; Khan et al., 2017; Lim et al., 2019) and thymol (Deb et al., 2011; Ferraz et al., 2013; Chauhan et al., 2018; Balan et al., 2021) in different concentration ranges well. However, some studies have demonstrated the action of these compounds, in a negative way, in some normal cells, such as human fibroblast cells (WS-1; IC_50_ of 138.1 ± 8.7 μM of carvacrol (Günes-Bayir et al., 2018); breast epithelial cells (fR2; IC_50_ of 86 μg/mL of thymol; (Pathania et al., 2013), normal lymphocyte (PBMC; IC_50_ > 25 μg/ml of thymol; (Ferraz et al., 2013); rat embryonic fibroblasts (mild toxicity of 20.94% after thymol 5, 30.5, 61, 122, 244 ng/mL; (Abed, 2011). Moreover, it has been shown that at concentrations of 0.5% (v/v) carvacrol and thymol exhibited a proliferative effect on normal human PBMC cells (Jaafari et al., 2007), and a lower concentration (10 μM) of carvacrol also caused a statistically significant proliferation of WS-1 cells (Günes-Bayir et al., 2018). In their most recent study, Günes-Bayir et al. (2020) reported that high doses of thymol can act on cancerous and healthy cells, while low doses seem to protect healthy cells but harm cancer cells. Moreover, genotoxic effects have been shown to be exhibited by carvacrol (Günes-Bayir et al., 2018) and thymol (Günes-Bayir et al., 2018), and it was also shown that a high concentration of carvacrol (460 µM) exhibited mutagenic and genotoxic effects causing DNA damage (Llana-Ruiz-Cabello et al., 2015). *In vivo*, Suntres et al. (2015) reported that the average lethal dose of carvacrol after intravenous administration in dogs was 310 mg/kg, for rats it was 810 mg/kg when administered orally and 80 and 73 mg/kg when injected intravenously or intraperitoneally, respectively. In mice, the lethal dose was 110–233.3 mg/kg, after inducing ataxia and drowsiness (Suntres et al., 2015). However, a phase I randomized clinical trial conducted in healthy subjects treated with carvacrol (1 or 2 mg/kg daily, p.o., during one month) revealed that this monoterpene did not lead to clinically significant changes, and did not cause any adverse effects, showing clinical safety and tolerability for this agent (Ghorani et al., 2021). Thus, the dose-response relationship must be considered in more detail, so that further studies in more advanced stages are developed to assess the antitumor effects of these compounds.

The methodological quality of the studies included revealed many items classified as “unclear” or “uncertain” indicating that the report—and presumably the experimental design—of these studies can be improved, especially with regard to the generation of the random sequence, the concealment of allocation and the blinding of researchers and evaluators, important factors in respect of a reliable result. Therefore, future studies need to improve their methodological quality, particularly in respect of risk of bias, in order to produce more reliable results. Even after almost two decades of research in this field, there has been no progress in research in humans, possibly due limitations of preclinical studies and the lack of knowledge about pharmacokinetics and toxicity of carvacrol and thymol. These factors limit the introduction of these compounds as a therapeutic option, and highlight the new for innovative studies to be undertaken.

Seven studies used a positive control to compare the antitumor effect of carvacrol (Jaafari et al., 2007; Maryam et al., 2015; Tayarani-Najaran et al., 2019) and/or thymol (Deb et al., 2011; Ferraz et al., 2013; Kang et al., 2016; Zeng et al., 2020) in their studies. *In vitro*, methotrexate and vincristine (0.5 µg/100 µL) (Jaafari et al., 2007), 5-fluorouracil (5-FU) (2.6 μg/μM) (Maryam et al., 2015), doxorubicin (0.5, 5, 10, and 20 μg/mL) (Tayarani-Najaran et al., 2019) (1.0 μg/mL) (Ferraz et al., 2013) and (2 μM) (Kang et al., 2016), camptothecin (5 μM) (Deb et al., 2011) and *in vivo*, doxorubicin (2 mg/kg) (Zeng et al., 2020), were the main substances used to compare the potential therapeutic effect of these monoterpenes. We emphasize here the importance of using a standard substance as a positive control as a parameter for comparing the effect of new candidates for cancer treatment.

In summary, we observed that more *in vivo* studies, particularly in respect of thymol, are needed. Further studies are required to help unravel and define the mechanisms of action of the two compounds against cancer cells, as well as studies that further explore their chemopreventive and anti-metastatic effects. It is of note that several studies suggested that signaling pathways (PI3K/AKT/mTOR and MAPKs) may be the main mechanism of action of these monoterpenoids, but more advanced studies are needed to elucidate this issue. There is also still a wide variation in the doses used, requiring the establishment of a consensus in respect of defining potentially effective and safe doses. In addition, further studies are also needed to clarify the effects of these two compounds on normal/healthy cells *in vitro,* and in appropriate *in vivo* models.

## Conclusion

The knowledge obtained through the reviewed studies provides strong evidence of the antitumor and antiproliferative activity promoted by carvacrol and thymol. However, there are still gaps regarding the standard or ideal dose, the exact mechanism of action and safety of the two compounds, revealing challenges for future studies. In addition, it was found through *in vitro* studies that carvacrol seems more potent than thymol, and was shown to have a greater cytotoxic effect for some cell lines. Moreover, further animal studies should be encouraged, as they have as yet made limited progress, and most of the current results are based on *in vitro* studies.

## Data Availability

The original contributions presented in the study are included in the article/Supplementary Material, further inquiries can be directed to the corresponding author.
