# Supplementary material for: Antitumor Effects of Carvacrol and Thymol: A Systematic Review
Source: Front Pharmacol. 2021 Jul 7;12:702487. doi: 10.3389/fphar.2021.702487 (PMC8293693; doi:10.3389/fphar.2021.702487)
Supplement: Supplementary file 1 [file DataSheet1.docx]

Supplementary Material

1. **Methods**
   1. **Search strategy**

Table S1: Search strategy used in Pubmed, Web of Science, Scopus and Lilacs databases.

| Data  Bases | Strategy | Results |
| --- | --- | --- |
| Pubmed | ((Drug Screening Assays, Antitumor) OR (Antitumor Drug Screening Assays) OR (Cancer Drug Test) OR (Drug Test, Cancer) OR (Antitumor Drug Screen) OR (Anti-Cancer Drug Screen) OR (Cell Transformation, Neoplastic) OR (Tumorigenic Transformation) OR (Transformation, Tumorigenic) OR (Neoplastic Cell Transformation) OR (Experimental Neoplasm) OR (Neoplasm, Experimental) OR (Antineoplastic Agent) OR (Antineoplastic Drug) OR (Antineoplastics) OR (Chemotherapeutic Anticancer Drug) OR (Antitumor Drug) OR (Cancer Chemotherapy Agents) OR (Cancer Chemotherapy Drugs) OR (Agents, Chemotherapeutic Anticancer) OR (Anticancer Agents) OR (Antitumor Agents) OR (Immunological Cytotoxicity Test) OR (Lymphocytotoxicity Tests, Antiglobulin-Augmented) OR (Microcytotoxicity Test) OR (Anti-Human Globulin Complement-Dependent Cytotoxicity Tests) OR (AHG-CDC Tests) OR (Anti-Human Globulin Complement-Dependent Cytotoxicity Test) OR (Cellular Proliferation) OR (Cell Multiplication) OR (Cell Growth in Number) OR (Tumor Cells, Cultured Neoplastic Cells, Cultured) OR (Cultured Neoplastic Cells) OR (Cell, Cultured Neoplastic) OR (Cells, Cultured Neoplastic) OR (Cultured Neoplastic Cell) OR (Neoplastic Cell, Cultured) OR (Cultured Tumor Cells) OR (Cell, Cultured Tumor) OR (Cells, Cultured Tumor) OR (Cultured Tumor Cell) OR (Tumor Cell, Cultured)) AND ((carvacrol) OR (5-Isopropyl-2-methylphenol) OR (Thymol) OR (5-methyl-2-propan-2-ylphenol)) | 594 |
| Web of Science | TS=((“Drug Screening Assays, Antitumor” OR “Antitumor Drug Screening Assays” OR “Cancer Drug Test” OR “Drug Test, Cancer” OR “Antitumor Drug Screen” OR “Anti-Cancer Drug Screen” OR “Cell Transformation, Neoplastic” OR “Tumorigenic Transformation” OR “Transformation, Tumorigenic” OR “Neoplastic Cell Transformation” OR “Experimental Neoplasm” OR “Neoplasm, Experimental” OR “Antineoplastic Agent” OR “Antineoplastic Drug” OR “Antineoplastics” OR “Chemotherapeutic Anticancer Drug” OR “Antitumor Drug” OR “Cancer Chemotherapy Agents” OR “Cancer Chemotherapy Drugs” OR “Agents, Chemotherapeutic Anticancer” OR “Anticancer Agents” OR “Antitumor Agents” OR “Immunological Cytotoxicity Test” OR “Lymphocytotoxicity Tests, Antiglobulin-Augmented” OR “Microcytotoxicity Test” OR “Anti-Human Globulin Complement-Dependent Cytotoxicity Tests” OR “AHG-CDC Tests” OR “Anti-Human Globulin Complement-Dependent Cytotoxicity Test” OR “Cellular Proliferation” OR “Cell Multiplication” OR “Cell Growth in Number” OR “Tumor Cells, Cultured Neoplastic Cells, Cultured” OR “Cultured Neoplastic Cells” OR “Cell, Cultured Neoplastic” OR “Cells, Cultured Neoplastic” OR “Cultured Neoplastic Cell” OR “Neoplastic Cell, Cultured” OR “Cultured Tumor Cells” OR “Cell, Cultured Tumor” OR “Cells, Cultured Tumor” OR “Cultured Tumor Cell” OR “Tumor Cell, Cultured”)) AND TS=((“carvacrol” OR “5-Isopropyl-2-methylphenol” OR “Thymol” OR “5-methyl-2-propan-2-ylphenol”)) | 169 |
| Scopus | TITLE-ABS-KEY (("Drug Screening Assays, Antitumor"  OR  "Antitumor Drug Screening Assays" OR "Cancer Drug Test" OR "Drug Test, Cancer" OR "Antitumor Drug Screen" OR*“*Anti-Cancer Drug Screen" OR "Cell Transformation, Neoplastic" OR "Tumorigenic Transformation" OR "Transformation, Tumorigenic" OR "Neoplastic Cell Transformation" OR "Experimental Neoplasm" OR "Neoplasm, Experimental" OR  "Antineoplastic Agent" OR "Antineoplastic Drug" OR "Antineoplastics" OR "Chemotherapeutic Anticancer Drug" OR "Antitumor Drug" OR "Cancer Chemotherapy Agents" *OR* "Cancer Chemotherapy Drugs" OR "Agents, Chemotherapeutic Anticancer" OR "Anticancer Agents" OR "Antitumor Agents"*OR*"Immunological Cytotoxicity Test" OR "Lymphocytotoxicity Tests, Antiglobulin-Augmented" OR "Microcytotoxicity Test" OR "Anti-Human Globulin Complement-Dependent Cytotoxicity Tests" OR "AHG-CDC Tests" OR "Anti-Human Globulin Complement-Dependent Cytotoxicity Test" OR "Cellular Proliferation" OR "Cell Multiplication" OR "Cell Growth in Number" OR "Tumor Cells, Cultured Neoplastic Cells, Cultured" OR "Cultured Neoplastic Cells" OR "Cell, Cultured Neoplastic" OR "Cells, Cultured Neoplastic" OR "Cultured Neoplastic Cell" OR "Neoplastic Cell, Cultured" OR "Cultured Tumor Cells" OR "Cell, Cultured Tumor" OR "Cells, Cultured Tumor" OR "Cultured Tumor Cell" OR "Tumor Cell, Cultured"*)* AND *(*"carvacrol" OR "5-Isopropyl-2-methylphenol" OR "Thymol" OR "5-methyl-2-propan-2-ylphenol"*))* | 194 |
| Lilacs | (tw: (Drug Screening Assays, Antitumor) OR (Antitumor Drug Screening Assays) OR (Cancer Drug Test) OR (Drug Test, Cancer) OR (Antitumor Drug Screen) OR (Anti-Cancer Drug Screen) OR (Cell Transformation, Neoplastic) OR (Tumorigenic Transformation) OR (Transformation, Tumorigenic) OR (Neoplastic Cell Transformation) OR (Experimental Neoplasm) OR (Neoplasm, Experimental) OR (Antineoplastic Agent) OR (Antineoplastic Drug) OR (Antineoplastics) OR (Chemotherapeutic Anticancer Drug) OR (Antitumor Drug) OR (Cancer Chemotherapy Agents) OR (Cancer Chemotherapy Drugs) OR (Agents, Chemotherapeutic Anticancer) OR (Anticancer Agents) OR (Antitumor Agents) OR (Immunological Cytotoxicity Test) OR (Lymphocytotoxicity Tests, Antiglobulin-Augmented) OR (Microcytotoxicity Test) OR (Anti-Human Globulin Complement-Dependent Cytotoxicity Tests) OR (AHG-CDC Tests) OR (Anti-Human Globulin Complement-Dependent Cytotoxicity Test) OR (Cellular Proliferation) OR (Cell Multiplication) OR (Cell Growth in Number) OR (Tumor Cells, Cultured Neoplastic Cells, Cultured) OR (Cultured Neoplastic Cells) OR (Cell, Cultured Neoplastic) OR (Cells, Cultured Neoplastic) OR (Cultured Neoplastic Cell) OR (Neoplastic Cell, Cultured) OR (Cultured Tumor Cells) OR (Cell, Cultured Tumor) OR (Cells, Cultured Tumor) OR (Cultured Tumor Cell) OR (Tumor Cell, Cultured)) AND (tw: (carvacrol) OR (5-Isopropyl-2-methylphenol) OR (Thymol) OR (5-methyl-2-propan-2-ylphenol)) | 213 |

# 3 Results

## 3.2 Overview of included studies

**Figure S1.** Distribution of the number of publications per year.

**Table S2:** Comparison between the IC_50_ of cells in vitro after 24 incubation with carvacrol and thymol.

| **REFERENCE** | **CELL TYPE** | **IC_50_ – Carvacrol (μM)** | **IC_50_ – Thymol**  **(μM)** |
| --- | --- | --- | --- |
| Horvathova; Turcaniova; Slamenova, (2007) | K-562 | 150-200 | 400-500 |
| Slamenová et al., (2007)  Özkan; Erdogan, (2011)  Yin et al., (2012)  Melušová et al., (2014)  Elshafie et al., (2017) | HepG2 | 350  353.46  400  425  319.5 | 400  399.53  -  -  1924.10 |
| Slamenová et al., (2007)  Llana-Ruiz-Cabello et al., (2014) | Caco-2 | 600  460 ± 3.6 | 700  - |
| Özkan; Erdoğan, (2012) | H1299 | 380 | 497 |
| Satooka; Kubo, (2012) | B16-F10 | 550 | 400 |
| Khan et al., (2017)  Luo et al., (2016) | DU 145 | 84.39  430.6 ± 21.9 | -  - |
| Günes-Bayir; Kocyigit; Güler, (2018) | AGS | 82.57 ± 5.58 | 75.63 ± 4.01 |
| Jamali et al., (2018)  Mari et al., (2020) | MCF-7 | 309.58  200 | 312.91  - |
| Jamali et al., (2018)  Baranauskaite et al., (2017) | MDA-MB 231 | 352.86  199 | 372.83  - |
| Potočnjak; Gobin; Domitrović, (2018) | HeLa | 556 ± 39 | - |
| Khan et al., (2019)  Luo et al., (2016) | PC-3 | 46.71  498.3 ± 12.2 | -  - |
| Elbe et al., (2020) | SKOV-3 | 322.50 | 316.08 |
| ***Mean ± S.E.M.*** | ***All*** | ***336.7 ± 35.0*** | ***527.1 ± 146.6*** |

**Table S3:** Isolated chemical compound.

| **Study** | **Compound, concentration** | **Source** | | **Purity (%)** | | | **Quality control reported** |
| --- | --- | --- | --- | --- | --- | --- | --- |
| Zeytinoglu; Incesu; Baser, (2003) (94) | Carvacrol, 1 – 150 μg/mL | Isolated from stem distillated essential oil of *Origanum onites L.* | 99% | | | Y - CG-MS | |
| Koparal; Zeytinoglu, (2003) (32) | Carvacrol, 100 – 1000 μM | Isolated from essential oil of *Origanum onites L.* | - | | | N | |
| Horváthová et al., (2006) (73) | Carvacrol, 25 - 900 μmol Thymol, 25 - 900 μmol | Fluka | - | | | N | |
| Karkabounas et al., (2006) (86) | Carvacrol, 10 – 4000 μM or  976 mg/mL | Sigma | - | | | N | |
| Horvathova; Turcaniova; Slamenova, (2007) (90) | Carvacrol, 200 – 1000 μM Thymol, 200 – 1000 μM | Fluka | Carvacrol: ≥ 97%  Thymol: ≥ 99% | | | N | |
| Jaafari et al., (2007) (87) | Carvacrol, 0.004 - 0.5 % v/v Thymol, 0.004 - 0.5 % v/v | - | - | | | N | |
| Slamenová et al., (2007) (96) | Carvacrol, 100 – 1000 μM Thymol, 150 - 1000 μM | Fluka | Carvacrol: ≥ 97%  Thymol: ≥ 99% | | | N | |
| Arunasree, (2010) (47) | Carvacrol, 20 – 100 μM | Sigma | 98% | | | N | |
| Akalin; Incesu, (2011) (95) | Carvacrol, 0.0002 - 0.1 mg/mL | - | - | | | N | |
| Mehdi et al., (2011) (49) | Carvacrol, 25 – 500 μg/mL | Sigma | 98% | | | N | |
| Özkan; Erdogan, (2011) (35) | Carvacrol, 20 – 200 μg/mL Thymol, 20 - 200 μg/mL | Carvacrol: Aldrich  Thymol: Sigma | Carvacrol: 98%  Thymol: 99,5% | | | N | |
| Abed, (2011) (77) | Thymol, 15, 30.5, 61,  122, 244 ng/mL | Extracted from the leaves of *Thymus vulgaris* | - | | | N | |
| Chang et al., (2011) (89) | Thymol, 100, 200, 400, 600 μmol/L | Sigma | - | | | N | |
| Deb et al., (2011) (92) | Thymol, 5, 25, 50, 75, 100 μM | Sigma | - | | | N | |
| Hsu et al., (2011) (85) | Thymol, 200, 300, 400,  500, 600, 800 μM | Sigma | - | | | N | |
| Jaafari et al., (2012) (88) | Carvacrol, 0.05 - 1.25 μM Thymol, 0.05 - 1.25 μM | Sigma | - | | | N | |
| Liang; Lu, (2012) (80) | Carvacrol, 200 – 1000 μM | Sigma | - | | | N | |
| Özkan; Erdoğan, (2012) (34) | Carvacrol, 25 – 1800 μM  Thymol, 10 - 2000 μM | - | - | | | N | |
| Satooka; Kubo, (2012) (65) | Carvacrol, -  Thymol, 75, 150, 300,  600, 1200 μM | Aldrich | - | | | N | |
| Yin et al., (2012) (37) | Carvacrol, 0.05 - 0.4 mmol/L | Sigma | - | | | N | |
| Jayakumar et al., (2012) (97) | Carvacrol, 15 mg/kg | Sigma | - | | | N | |
| Ahmed et al., (2013) (99) | Carvacrol, 15 mg/kg | Sigma | - | | | N | |
| Liang et al., (2013) (53) | Carvacrol, 200 – 1000 μM | Sigma | - | | | N | |
| Ferraz et al., (2013) (66) | Thymol, 1.56 - 50 μg/mL | Sigma | ≥ 99.5% | | | N | |
| Pathania et al., (2013) (68) | Thymol, 10, 30,50,  70, 100 μg/mL | - | - | | | N | |
| Subramaniyan et al., (2014) (98) | Carvacrol, 15 mg/kg | Sigma | 99% | | | N | |
| Al-Fatlawi; Ahmad, (2014) (44) | Carvacrol, 140 – 450 μM | Sigma | - | | | - | |
| Aydin; Türkez; Keleş, (2014) (81) | Carvacrol, 10 – 400 mg/L | Sigma | - | | | N | |
| Llana-Ruiz-Cabello et al., (2014) (39) | Carvacrol, 100 – 2500 μM  Thymol, 100 – 2500 μM | - | - | | | N | |
| Melušová et al., (2014) (36) | Carvacrol, 25 – 1000 μM | Fluka | ≥ 97% | | | N | |
| Melušová; Jantová; Horváthová, (2014) (38) | Carvacrol, 100 – 600 μM | Fluka | ≥ 97% | | | N | |
| Chen et al., (2015) (83) | Carvacrol, 125 – 1000 μM | Sigma | - | | | N | |
| Fan et al., (2015) (40) | Carvacrol, 100 – 900 μmol/L | Sigma | - | | | N | |
| Maryam; Shakeri; Kiani, (2015) (62) | Carvacrol, 0,01 - 6 mg/mL | Sigma | 98% | | | N | |
| Bhakkiyalakshmi et al., (2016) (91) | Carvacrol, 10 – 200 μM | Sigma | **-** | | | N | |
| Dai et al., (2016) (54) | Carvacrol, 10 – 80 μM | Sigma | - | | | N | |
| Fitsiou et al., (2016) (198) | Carvacrol, 1 – 1000 μM Thymol, 1 - 1.000 μM | Sigma | - | | | N | |
| Luo et al., (2016) (57) | Carvacrol, 250 – 750 μM | Sigma | - | | | N | |
| Coccimiglio et al., (2016) (71) | Carvacrol, 0-250 μM  Thymol, 0-250 μM | Sigma | - | | | Y - GC-MS | |
| Kang et al., (2016) (139) | Thymol, 100, 200, 400 μM | Sigma | - | | | N | |
| Lee et al., (2016) (84) | Thymol, 0.1, 0.3, 1, 3, 10,  30, 100, 200 µM | Sigma | - | | | N | |
| Sivaranjani; Sivagam; Nalini, (2016) (100) | Carvacrol, 20, 40, 80 mg/kg | Sigma | - | | | N | |
| Baranauskaite et al., (2017) (46) | Carvacrol, 10^-5^ – 10^-2.5^ M | Sigma-Aldrich | > 98% | | | N | |
| Elshafie et al., (2017) (72) | Carvacrol, 0.01 - 0.25 μg/μL  Thymol, 0.06, 0.11, 0.22,  0.45, 0.90 μg/μL | - | - | | | N | |
| Horng et al., (2017) (56) | Carvacrol, 100 – 800 μM | Sigma | - | | | N | |
| Khan et al., (2017) (55) | Carvacrol, 10 – 500 μM | Sigma | - | | | N | |
| Chauhan et al., (2017) (74) | Thymol, 100, 150, 200 μg/mL | Sigma | 99,9% | | N | | |
| Li et al., (2017) (78) | Thymol, 25, 50, 100, 150 μM | Sigma | - | | | N | |
| Yeh et al., (2017) (79) | Thymol, 100, 300, 500,  700, 900 μM | Sigma | - | | | N | |
| Al-Fatlawi, (2018) (51) | Carvacrol, 140 – 450 μM | Sigma | - | | | N | |
| De La Chapa et al., (2018) (69) | Thymol, 200 - 800 µM  or 4.3 mM | Sigma | - | | | N | |
| Govindaraju; Arulselvi, (2018) (67) | Carvacrol, 3.906 – 1000 μg/mL | Isolated from essential oil of *Coleus Aromaticus* | - | | | N | |
| Günes-Bayir; Kocyigit; Güler, (2018) (60) | Carvacrol, 10 – 600 µM  Thymol, 10 – 600 µM | Sigma | - | | | N | |
| Günes-Bayir et al., (2018) (61) | Carvacrol, 10 – 600 µM | Sigma | - | | | N | |
| Jamali et al., (2018) (42) | Carvacrol, 10 – 200 μg/mL | - | - | | | N | |
| Jung; Kim; Lee, (2018) (33) | Carvacrol, 30 – 300 μM | Sigma | - | | | N | |
| Lim et al., (2018) (64) | Carvacrol, 50 – 300 μM | Sigma | - | | | N | |
| Potočnjak; Gobin; Domitrović, (2018) (50) | Carvacrol, 100 – 800 µM | Sigma | 98% | | | N | |
| Heidarian; Keloushadi, (2019) (58) | Carvacrol, 100 – 800 μM | Sigma | - | | | N | |
| Khan et al., (2019) (59) | Carvacrol, 10 – 500 μM | Sigma | - | | | N | |
| Tayarani-Najaran et al., (2019) (45) | Carvacrol, 31.2 - 500 μg/mL | - |  | | |  | |
| Trindade et al., (2019) (201) | Carvacrol, 25 – 200 μg/mL | Sigma | 98% | | | N | |
| Li, Zhao, Bao; (2019) (101) | Carvacrol, information not provided by the author | Western Chemical  Technology Co | ≥ 99,0% | | |  | |
| Seresht et al., (2019) (76) | Thymol, 5, 10, 20, 30, 40,  50, 75, 100 g/mL | - |  | | |  | |
| Thapa et al., (2019) (75) | Thymol, 62.5, 125, 250, 500,  750, 1000 ppm | Agolin | > 98% | | | N | |
| Elbe et al., (2020a) (21) | Thymol, 100, 200, 400,  600, 800 µM | Sigma | - | | | N | |
| Zeng et al., (2020) (103) | Thymol, 10, 20, 40,  80, 120 µg/mL  or 75 mg/kg and 150 mg/kg | Extracted from *T. vulgaris* |  | | |  | |
| Pakdemirli et al., (2020) (41) | Carvacrol, 25 - 200 μM | Sigma | - | | | N | |
| Günes-Bayir et al., (2020) (63) | Thymol, 0-600 μM | Sigma | - | | | N | |
| Mari et al., (2020) (48) | Carvacrol, 25 – 250 μmol/L | Sigma | 98% | | | N | |
| Elbe et al., (2020) (52) | Carvacrol, 100, 200,  400, 600 μM  Thymol, 100, 200,  400, 600 μM | Sigma | - | | | N | |
| Rojas-Armas et al., (2020) (102) | Carvacrol, 50, 100, 200 mg/kg | Sigma | - | | | N | |
| Kocal; Pakdemirli, (2020) (82) | Carvacrol, 12.5, 25, 50 µM | - | - | | | N | |
| Li et al., (2021) (43) | Carvacrol, 25–500 μM | Sigma | - | | | N | |
| Bouhtit et al., (2021) (93) | Carvacrol, 100, 200,  300, 400 μM  Thymol, 25, 50,  100 μM | Sigma | Carvacrol: 99,9%  Thymol: 98,5% | | | N | |
| Balan et al., (2021) (70) | Thymol, 25–200 μg/mL | Alfa Aesar | - | | | N | |
| Hassan et al., (2021) (104) | Thymol, 20 mg/kg | Sigma | - | | | N | |
